# OsMYB7 determines leaf angle at the late developmental stage of lamina joints in rice

**DOI:** 10.3389/fpls.2023.1167202

**Published:** 2023-04-14

**Authors:** Suk-Hwan Kim, Jungwon Yoon, Hanna Kim, Sang-Ji Lee, Taehoon Kim, Kiyoon Kang, Nam-Chon Paek

**Affiliations:** ^1^ Department of Agriculture, Forestry and Bioresources, Plant Genomics and Breeding Institute, Research Institute of Agriculture and Life Sciences, Seoul National University, Seoul, Republic of Korea; ^2^ Division of Life Sciences, Incheon National University, Incheon, Republic of Korea

**Keywords:** auxin, cellulose, leaf angle, lignin, OsMYB7, rice

## Abstract

Leaf angle shapes plant architecture, allowing for optimal light interception to maximize photosynthesis and yield, and therefore is a crucial agronomic trait. Here, we show that the rice (*Oryza sativa* L.) R2R3-type MYB transcription factor OsMYB7 determines leaf angle in a developmental stage-specific manner. *OsMYB7*-overexpressing lines produced wide-angled leaves and *osmyb7* knockout mutants exhibited erect leaves. This phenotype was restricted to the lamina joints at the late developmental stage. In agreement with these observations, *OsMYB7* was preferentially expressed in the lamina joints of post-mature leaves. Since OsMYB7 homologs are transcriptional repressors of lignin biosynthesis, we examined whether OsMYB7 might inhibit thickening of secondary cell walls. Although OsMYB7 repressed lignin biosynthesis, it enhanced thickening of sclerenchyma cell walls by elevating cellulose contents at the lamina joints. Furthermore, we found that OsMYB7 affects endogenous auxin levels in lamina joints, and the adaxial cells of lamina joints in *OsMYB7*-overexpressing lines and *osmyb7* knockout mutants exhibited enhanced and reduced elongation, respectively, compared to the wild type. These results suggest that OsMYB7 promotes leaf inclination partially through decreasing free auxin levels and promoting cell elongation at the adaxial side of lamina joints.

## Introduction

Leaf angle, the angle formed between the leaf blade and the culm, is a major agronomic trait in rice (*Oryza sativa* L.) (Donald, 1968). Indeed, erect leaves improve penetration of sunlight into dense fields, thus enhancing photosynthetic efficiency of the canopy and allowing higher planting density for increased grain production (Wang J. et al., 2020). Plant hormones are the primary factors controlling leaf inclination (Xu et al., 2021). Among them, brassinosteroids (BRs) play a dominant role in determining leaf angle, as most of the mutants with altered leaf angles are associated with malfunctions of BR metabolism or signaling. For example, mutants with elevated BR contents or enhanced BR signaling exhibited increased angles of the lamina joints, whereas those with reduced BR contents or diminished BR signaling exhibited erect leaves (Yamamuro et al., 2000; Mori et al., 2002; Wang et al., 2002; Tanabe et al., 2005; Sakamoto et al., 2006; Jang, 2017). These studies also revealed that BRs accelerate elongation and/or division of the adaxial parenchyma cells at lamina joints, indicating that BRs positively regulate leaf inclination.

Indole-3-acetic acid (IAA), the most abundant naturally occurring auxin, is also a crucial plant hormone that controls leaf angle (Li et al., 2020). In contrast to BRs, IAA negatively regulates leaf inclination, based on the observations that higher IAA contents result in upright leaves while lower IAA causes horizontal leaves (Kim et al., 2013; Yoshikawa et al., 2014). IAA homeostasis is coordinately modulated by its biosynthesis and conjugation. Most IAA is synthesized *via* two enzymatic steps involving tryptophan aminotransferase (TAA) and flavin monooxygenase (YUCCA) family members. In addition, conjugation of IAA with amino acids, such as aspartate and glutamate, is mainly catalyzed by GRETCHEN HAGEN 3 (GH3) family members and effectively reduces the free IAA concentration (Wang et al., 2018; Hayashi et al., 2021). To date, at least five rice IAA homeostatic genes (*OsTAA1*, *OsGH3-1*, *OsGH3-2*, *OsGH3-5*, and *OsGH3-13*) have been identified as involved in regulating leaf angle (Zhang et al., 2009; Du et al., 2012; Zhao et al., 2013; Yoshikawa et al., 2014; Zhang et al., 2015). When *OsTAA1* was mutated or *OsGH3* genes were overexpressed, the resulting deficiency in IAA promoted the expansion or division of parenchyma cells at the adaxial side of lamina joints, thereby increasing leaf angles. In addition to these IAA homeostasis genes, several auxin signaling genes, such as *AUXIN-RESPONSE FACTOR 19* (*OsARF19*) and *LEAF INCLINATION 3* (*LC3*), affect leaf inclination by directly regulating the expression of *OsGH3*s (Zhang et al., 2015; Chen et al., 2018).

Besides phytohormones, lignocellulose, a main structural component of the secondary cell wall (SCW), modulates leaf angle through determining the mechanical strength of lamina joints; lignocellulose-deficient mutants exhibited wide leaf angles and mutants with increased lignocellulose contents showed an erect-leaf phenotype (Sun et al., 2020; Wang R. et al., 2020; Huang et al., 2021). For instance, *OVATE FAMILY PROTEIN 6* (*OsOFP6*) knockout rice mutants displayed much wider leaf angles due to the lack of lignocellulose at their lamina joints in a BR-independent manner (Sun et al., 2020). In another case, OsARF6 and OsARF17 were shown to maintain SCW-mediated mechanical strength for supporting leaf weight: the *osarf6 osarf17* double mutant exhibited a horizontal leaf angle phenotype due to lignocellulose deficiency at the lamina joints (Huang et al., 2021). Recently, several key transcription factors, which are predicted to downregulate lignin biosynthesis during lamina joint development, were proposed to contribute to leaf inclination: the knockout lines of the corresponding genes, such as Os04g0549700, Os02g0656600, Os03g0182800, and Os07g0674800, had more lignin contents in the sclerenchyma cells of their lamina joints, thereby exhibiting upright leaves (Wang R. et al., 2020).

Several MYB transcription factors regulating biosynthetic programs of SCW components have been identified in plants (Miyamoto et al., 2020). Although the functions of the MYB transcription factors in deposition of lignin, cellulose, and xylan have been extensively studied, additional possible role in phytohormone metabolism or cell growth is not well documented. *OsMYB7* encodes a MYB transcriptional repressor with cell wall–associated functions and in this study, we investigated the plants which overexpressed or knockout of *OsMYB7*. Our findings demonstrate that OsMYB7 increases leaf angle: two independent *OsMYB7* overexpressors displayed wide leaf angles, whereas *osmyb7-1* knockout mutant showed narrow leaf angles. OsMYB7 inhibited lignin accumulation in the lamina joints. However, further studies revealed that OsMYB7 slightly increases the thickness of the SCW, a primary determinant of mechanical strength, by promoting cellulose deposition. Our results suggest that the alteration in leaf angle may not be primarily due to OsMYB7-mediated modulation of the SCW components, although we cannot exclude the possibility that the leaf angle phenotypes of *OsMYB7* overexpressors and *osmyb7-1* knockout mutant are due to altered levels of phenylpropanoids. We also found that the contents of free auxin in lamina joints were changed in *OsMYB7*-overexpressing lines and *osmyb7* knockout mutants and that the cell size at the adaxial side of lamina joints was altered in these plants compared to that of WT. Thus, we propose that OsMYB7 increases rice leaf angles partially by decreasing auxin contents and accelerating parenchyma cell elongation at the adaxial side of lamina joints. Collectively, our findings provide a new perspective on the biological functions of cell wall-associated MYB transcription factors and offer a potential biotechnological avenue for breeding new gene-edited rice varieties with erect leaves for high-density cropping.

## Materials and methods

### Plant materials and growth conditions

The activation-tagged T-DNA insertion line of *OsMYB7*, designated *OsMYB7-OE1* (PFG_4A-03610), was obtained from the Salk Institute Genomics Analysis Laboratory (http://signal.salk.edu/cgi-bin/RiceGE) (Jeon et al., 2000; Jeong et al., 2002). Plants of *OsMYB7-OE1*, *OsMYB7-OE2*, *osmyb7-1*, and the parental *japonica* cultivar ‘Dongjin’ (referred to as wild type; WT) rice (*Oryza sativa*) were cultivated under natural long day conditions (around 14 h light per day) in a paddy field (Suwon, Republic of Korea, 37°N latitude). The seedlings were initially grown for one month in a greenhouse after sowing on seed beds, and subsequently transplanted into the paddy field following standard agricultural practices for Korean rice varieties.

### Vector construction and rice transformation

To generate the *OsMYB7-OE2* transgenic line, the full-length coding sequence of *OsMYB7* was amplified by PCR from cDNA obtained from total RNA extracted from lamina joints of WT as template and gene-specific primers (**Supplementary Table S4**). The PCR product was subcloned into the entry vector pCR™8/GW/TOPO^®^ (Invitrogen, Carlsbad, CA, USA) and then transferred into the gateway-compatible binary destination vector pMDC32 (Curtis and Grossniklaus, 2003) by performing an LR recombination reaction using a Gateway™ LR Clonase™ II Enzyme Mix (Invitrogen). To generate the *osmyb7-1* mutant, an *OsMYB7*-specific 20-nt spacer sequence, TGTCAGGTGGTCTCTGATCG, was designed using the CRISPRdirect software (https://crispr.dbcls.jp/) (Naito et al., 2015) and subcloned into the single guide RNA expression cassette of the pOs-sgRNA entry vector (Miao et al., 2013). The resulting cassette was then transferred into the pH-Ubi-cas9-7 destination vector containing a *Cas9* expression cassette (Miao et al., 2013) through LR reaction.

The plasmid constructs were introduced into *Agrobacterium tumefaciens* strain LBA4404 (Ooms et al., 1982) *via* the freeze-thaw method (Höfgen and Willmitzer, 1988). Mature seed embryos of WT were used to generate calli, which were then transformed with the *Agrobacterium*-mediated transformation protocol described by Jeon et al. (2000). The transformed calli were selected on 2N6 medium supplemented with 50 mg L^-1^ hygromycin (Duchefa Biochemie, Haarlem, the Netherlands), and the regenerated plants were confirmed as transgenic rice.

### Transactivation and trans-repression assays

For transactivation assay, we cloned the full-length coding sequence of *OsMYB7* into the vector pGBKT7 (BD Biosciences Clontech) between *Sal*I and *Not*I sites, and that of *OsbHLH079* into the same vector between *Eco*RI and *Not*I sites. For trans-repression assay, the activation domain of GAL4 transcription factor in the vector pGADT7 (BD Biosciences Clontech) was cloned in frame between *Nco*I and *Eco*RI sites in the vector pGBKT7 (BD Biosciences Clontech) to generate the vector rGAL4, as previously described (Mathew et al., 2016). Next, we cloned the full-length coding sequences of *OsMYB7* and *ONAC026* into the vector rGAL4 between *Sal*I and *Not*I sites. All the resulting constructs were transformed into the *Saccharomyces cerevisiae* strain AH109. Subsequently, yeast β-galactosidase liquid assays were performed according to the Yeast Protocols Handbook (BD Biosciences Clontech) using chlorophenol red-β-D-galactopyranoside (CPRG; Roche, Basel, Switzerland) as substrate. Briefly, we incubated yeast extracts in 8 mM CPRG (Roche) for 30 min at 30°C in the dark and measured the absorbance of extracts at a wavelength of 574 nm using a UV/VIS spectrophotometer (PowerWave X, BioTek, Winooski, USA). Primers details are provided in **Supplementary Table S4**.

### Subcellular localization

The full-length coding sequence of *OsMYB7* subcloned into the entry vector pCR™8/GW/TOPO^®^ (Invitrogen) was transferred into the Gateway-compatible plant destination vector pEarleyGate 104 (Earley et al., 2006) by the LR reaction. The resulting vector was introduced into onion (*Allium cepa*) epidermal cells by particle bombardment with a Biolistic PDS-1000/He instrument (Bio-Rad, Hercules, CA, USA). After bombardment, the onion epidermal layers were incubated on Murashige and Skoog (MS) medium (pH 5.7) for 16 h at 22°C in the dark. The nuclei were then stained with 300 nM of 4’,6-diamidino-2-phenylindole (DAPI; Invitrogen) in phosphate-buffered saline (PBS) for 3 min in darkness before observation. Fluorescence emission was analyzed using a Leica TCS SP8 X confocal laser scanning microscope (Leica Microsystems, Wetzlar, Germany) with the following parameters: excitation at 458 nm and emission at 514 nm for YFP, and excitation at 405 nm and emission at 488 nm for DAPI.

### Gene expression analysis (qPCR)

Total RNAs from rice plants were extracted using a MG Total RNA Extraction Kit (MGmed, Seoul, Republic of Korea) according to the manufacturer’s instructions and subjected to reverse transcription with an Oligo(dT)_15_ Primer (Promega, Madison, WI, USA) and M-MLV Reverse Transcriptase (Promega) to generate first-strand cDNAs. The resulting product mixtures were diluted with distilled water by a factor of four. Quantitative PCR (qPCR) was performed with a GoTaq^®^ qPCR Master Mix (Promega) using a LightCycler^®^ 480 System (Roche) as described in the instruction manual. The reaction mix of a 20-μl final volume for qPCR was prepared by combining the 2 μl of first-strand cDNA mixture, 10 μl of GoTaq^®^ qPCR Master Mix, 0.4 μl of 10 μM forward primer, 0.4 μl of 10 μM reverse primer, and 7.2 μl of nuclease-free water. The gene-specific primers are listed in **Supplementary Table S4**, and the qPCR conditions were as follows: 95°C for 2 min followed by 45 cycles of 95°C for 15 sec and 60°C for 1 min. Data obtained by qPCR were analyzed using the 2^–ΔΔCT^ method (Livak and Schmittgen, 2001) with *GAPDH* as a reference for normalization (Jain et al., 2006).

### Histochemical detection of GUS enzymatic activity

The lamina joints of *OsMYB7-OE1* carrying a *β-glucuronidase* (*GUS*) reporter gene driven by the *OsMYB7* promoter (**Supplementary Figure S3A**) were subjected to histochemical GUS staining according to the previously described method (Jefferson et al., 1987) with some modifications. Lamina joints at developmental stages S3, S4, and S5 were sampled and vacuum-infiltrated with a X-Gluc reaction buffer containing 100 mM sodium phosphate buffer (pH 7.0), 10 mM EDTA, 0.1% Triton X-100, 0.5 mM potassium ferricyanide, 0.5 mM potassium ferrocyanide, and 1 mM X-Gluc (Sigma-Aldrich, Saint Louis, MO, USA) for 30 min in darkness, followed by overnight incubation at 37°C under dark conditions. The GUS histochemical staining buffer was then replaced with 70% (v/v) aqueous ethanol to remove chlorophylls. After complete decolorization, images of lamina joints were capture with a digital camera, and transverse hand-cut sections of lamina joints were subsequently photographed using a stereo microscope (SteREO Discovery.V12, Carl Zeiss Microscopy GmbH, Jena, Germany) with dark filter.

### Phloroglucinol-HCl staining and calcofluor white staining

The lamina joints of rice plants were collected at 30 DAH and vacuum-infiltrated immediately in FAA solution (70% [v/v] aqueous ethanol, glacial acetic acid, and formalin in a ratio of 90:5:5 v/v/v) for 30 min, followed by overnight incubation at room temperature with gentle rotation. Hand-cut cross sections were prepared from the fixed lamina joints and washed twice with 70% (v/v) aqueous ethanol for 30 min each with gentle inversion. For histochemical observation of lignin, phloroglucinol-HCl staining was conducted according to a standard protocol (Halpin et al., 1998) with some modifications. Briefly, the sections were immersed in 1% (w/v) solution of phloroglucinol (Sigma-Aldrich) in 70% (v/v) aqueous ethanol for 5 min and then treated with 18% (v/v) aqueous HCl for 5 min before being observed with a stereo microscope (SteREO Discovery.V12, Carl Zeiss Microscopy GmbH). To visualize deposited cellulose, calcofluor white staining was carried out as described previously (Hageage and Harrington, 1984) with a little modification. Briefly, each section was stained with one drop of calcofluor white stain (Sigma-Aldrich) and one drop of 10% (w/v) aqueous potassium hydroxide for 2 min and examined using an inverted phase contrast fluorescence microscope (Axio Observer Z1, Carl Zeiss Microscopy GmbH) under DAPI filter.

### Preparation of destarched alcohol-insoluble cell wall residues

The isolation of dsAIRs for the quantification of lignins and celluloses was performed, following the procedure described previously (Zhang et al., 2019) with some modifications. Around 1 g of lamina joints from rice plants at 30 DAH was ground in liquid nitrogen and lyophilized using a freeze dryer (Bondiro, ilShin^®^ Lab Co. Ltd., Yang-Ju, Republic of Korea) for 48 h. Approximately 150 mg of each freeze-dried sample was weighed into a 5-mL snap-cap centrifuge tube and dissolved sequentially with 3 ml of 70% (v/v) aqueous ethanol for two times, 3 ml of chloroform/methanol (1:1 v/v) for one time, and 3 ml of acetone for one time, followed by centrifugation at 1,500 g for 10 min each. The resultant pellet was transferred to a 2 ml screw cap microcentrifuge tube and dried in an oven at 35°C until completely dry. The dried sample was then resuspended in 1.5 ml of 0.1 M sodiumacetate buffer (pH 5.0) and incubated in a heat block for 20 min at 80°C. After cooling on ice, the following agents were added: 35 μl of 0.01% (w/v) sodiumazide in 0.1 M sodiumacetate buffer (pH 5.0), 35 μl of 50 μg/ml amylase (α-Amylase from *Bacillus* sp., Sigma-Aldrich) in distilled water, and 17 μl of pullulanase (Pullulanase microbial, Sigma-Aldrich). Following overnight incubation in a shaking incubator at 37°C with gentle rpm, the suspension was heated for 10 min using a heat block at 100°C to terminate digestion. The solubilized starch in supernatant was then discarded after centrifugation at 1,500 g for 10 min. The resultant pellet was washed with 1.5 ml of distilled water for three times and 0.5 ml of acetone for two times, followed by centrifugation at 1,500 g for 10 min each. The remaining solvent was totally evaporated in an oven at 35°C. The dried material, dsAIR, was then subjected to the acetyl bromide assay for lignin or anthrone assay for cellulose as described below.

### Lignin quantification

To measure the lignin content in dsAIR, an acetyl bromide assay was conducted as previously described (Moreira-Vilar et al., 2014) with a little modification. The 20 mg of dsAIR was weighed into a 5 ml screw cap centrifuge tube and dissolved with 500 μl of 25% (v/v) acetyl bromide (Sigma-Aldrich) in glacial acetic acid for 1 h using an oven at 70°C. After cooling on ice, the following reagents were added successively to the suspension: 0.9 ml of 2 M aqueous NaOH, 0.1 ml of 5 M aqueous hydroxylamine-HCl, and 3.5 ml of glacial acetic acid. The absorbance of each supernatant containing solubilized lignin was measured at a wavelength of 280 nm (Su et al., 2005) using a UV/VIS spectrophotometer (PowerWave X, BioTek) after centrifugation at 1,400 g for 10 min. The content of lignin was subsequently calculated using a standard curve generated with alkali lignin (Sigma-Aldrich).

### Cellulose quantification

To quantify cellulose, an anthrone assay was performed after the isolation of crystalline cellulose from dsAIR, following a previously described protocol (Updegraff, 1969) with slight modifications. The 2-3 mg of dsAIR was weighed into a 2 ml screw cap microcentrifuge tube and treated with 250 μl of 2 M trifluoroacetic acid (Sigma-Aldrich) at 121°C for 2 h using a heat block. After cooling on ice, the supernatant was replaced with 1 ml of isopropanol after centrifugation at 11,000 g for 10 min. The sample was then centrifuged at 11,000 g for 10 min, and the supernatant was discarded. The remaining solvent was exhaustively removed using an oven at 35°C. The 1 ml of Updegraff reagent (glacial acetic acid: nitric acid: DW, 8:1:2 v/v/v) was added to the TFA pellet, and the suspension was heated in a heat block at 100°C for 1 h, followed by cooling on ice. The pellet, crystalline cellulose, was obtained after centrifugation at 11,000 g for 15 min and washed once with 1.5 ml of distilled water and three times with 1.5 ml of acetone, followed by centrifugation at 11,000 g for 10 min each. The resultant sample was incubated in an oven at 35°C for 16 h and then completely hydrolyzed into glucose by being treated with 175 μl of 72% (w/w) aqueous sulfuric acid (Alfa Aesar, Ward Hill, MA, USA) at room temperature for 90 min. After the dilution with 825 μl of distilled water, the suspension was centrifuged at 11,000 g for 5 min. The glucose content in resultant supernatant was analyzed using the colorimetric anthrone assay. The 10 μl of supernatant was mixed with 90 μl of distilled water and 200 μl of anthrone reagent in a 96-well polystyrene microtiter plate, followed by incubation in an oven at 80°C for 30 min. The standards prepared with D-(+)-glucose (Sigma-Aldrich) were also subjected to the anthrone assay on the same plate. Absorbance of each sample at 625 nm was read using a UV/VIS spectrophotometer (PowerWave X, BioTek), and the contents of cellulose were calculated based on a standard curve.

### Transmission electron microscopy

Sample preparation for TEM was conducted essentially following the conventional method (Inada et al., 1998) but with the help of microwave irradiation using a microwave tissue processor (hereafter termed microwave) (PELCO BioWave^®^ Pro+, Ted Pella, Redding, CA, USA) as previously described (Mowery and Bauchan, 2018) with some modifications. The collars from rice plants were harvested and vacuum-infiltrated in modified Karnovsky’s fixative (2% [w/v] glutaraldehyde and 2% [w/v] paraformaldehyde in 50 mM sodium cacodylate buffer, pH 7.2) for 30 min, followed by overnight incubation at 4°C. After being rinsed three times with 50 mM sodium cacodylate buffer (pH 7.2) at 4°C for 5 min each, the samples were post-fixed by 1% (w/v) osmium tetroxide in 50 mM sodium cacodylate buffer, pH 7.2, using the microwave (2 min On, 2 min Off, 2 min On, 2 min Off, 4 min On, 2 min Off, and 4 min On; 100 W; *T*_max_ = 30°C). Post-fixed specimens were then briefly washed three times with distilled water at room temperature and subjected to microwave-assisted *en bloc* staining with 0.5% (w/v) uranyl acetate (1 min On, 1 min Off, and 1 min On; 100 W; *T*_max_ = 30°C). The dehydration of samples was performed in the microwave with increasing concentrations of ethanol as follows: single changes in 30%, 50%, 70%, 80%, and 90% ethanol (all v/v) followed by three changes in 100% ethanol (40 s On; 150 W; *T*_max_ = 35°C each). The dehydrated specimens were subsequently incubated twice in propylene oxide under microwave irradiation (40 s On; 150 W; *T*_max_ = 35°C each) and gradually infiltrated with a graded series of increasing concentrations of Spurr’s resin in propylene oxide (once with 30%, 50%, and 80% Spurr’s resin each, followed by two times with 100% Spurr’s resin; all v/v) using the microwave (15 min On; 200 W; *T*_max_ = 40°C each). Next, samples were embedded in pure Spurr’s resin and finally polymerized in an oven at 70°C for 24 h. The specimens in the resulting resin blocks were thinly sectioned (70 nm) using an ultramicrotome (EM UC7, Leica Microsystems) with a Diatome diamond knife, mounted on Formvar-coated copper grids (EMS, Hatfield, PA, USA), and post-stained with 2% (w/v) uranyl acetate for 10 min and Reynolds’ lead citrate for 10 min at room temperature before being observed with a transmission electron microscope (Talos L120C, FEI, Czech Republic) operated at 120 kV.

### LC-ESI-MS/MS analysis

The contents of IAA, IAA-Asp, and IAA-Glu were measured following previously described procedures (Matsuda et al., 2005) with some modifications. Approximately 2 g of lamina joints from rice plants was homogenized with a mortar and pestle in liquid nitrogen and lyophilized using a freeze dryer (Bondiro, ilShin^®^ Lab Co. Ltd.) in darkness for 72 h. Around 500 mg of freeze-dried sample was weighed into a 10-mL light safe centrifuge tube and dissolved in 10 mL of 80% (v/v) aqueous acetone containing 2.5 mM diethyl dithiocarbamate using an ultrasonic bath (Powersonic 420, Hwashin Tech Co. Ltd., Gwangju, Republic of Korea) at 0°C for 2 h, followed by gentle rotation at 4°C for 4 h. After centrifugation at 3,500 g for 15 min at 4°C, the supernatant was transferred to a 50-mL light safe centrifuge tube. The extraction procedure was repeated once with the same sample, and the resulting supernatant was combined with the first supernatant in the 50-mL light safe centrifuge tube. As an internal standard, 0.2 nmol of [phenyl-^13^C_6_]-indole-3-acetic acid (Cambridge Isotope Laboratories, Andover, MA, USA) was added at the first step of extraction. The combined extract was then passed through s 0.2-μm PTFE membrane filter, and the solvent was completely evaporated in a vacuum. The residue was subsequently resuspended in 500 μL of 10% (v/v) aqueous methanol containing 1% (v/v) acetic acid and subjected to LC-ESI-MS/MS analysis: a Dionex UltiMate 3000 system (Thermo Fisher Scientific, CA, USA) coupled to a triple quadrupole mass spectrometer (TSQ Altis™, Thermo Fisher Scientific) was used for the analysis. A 10-μl aliquot of the sample was injected onto a Cadenza CD-C18 column (75 × 2.0 mm, 3 μm; Imtakt, Kyoto, Japan) and eluted using a mixture of methanol: 0.1% aqueous acetic acid (gradient from 10:90 to 90:10 in 13 min) with a flow rate of 200 μl/min at 30°C. The triple quadrupole mass spectrometer was equipped with an electrospray interface and operated using the multiple reaction monitoring (MRM) in positive ion mode, and the transitions from precursor to product ions for each compound were monitored as follows: [phenyl-^13^C_6_]-IAA, *m*/*z* of 182.1 to 136.0; IAA, *m*/*z* of 176.1 to 130.0; IAA-Asp, *m*/*z* of 291.1 to 130.0; IAA-Glu, *m*/*z* of 305.1 to 130.0. The levels of IAA, IAA-Asp, and IAA-Glu in each sample were calculated using corresponding calibration curves generated from each external standard. The standard for IAA was purchased from Sigma-Aldrich, and those for IAA-Asp and IAA-Glu were bought from Biosynth Carbosynth^®^ (Compton, Berkshire, UK).

### Scanning electron microscopy

Sample preparation for SEM was performed according to a method described previously (Bozzola and Kuo, 2007) with slight modifications. Approximately 1-cm lamina joint segments, containing leaf blade, collar, and leaf sheath, were hand-sectioned longitudinally and immersed overnight in modified Karnovsky’s solution (2% paraformaldehyde and 2% glutaraldehyde in 50 mM sodium cacodylate buffer, pH 7.2) at 4°C. The samples were then washed in three changes of 50 mM sodium cacodylate buffer, pH 7.2, at 4°C for 10 min each, post-fixed with 1% osmium tetroxide in 50 mM sodium cacodylate buffer (pH 7.2) at 4°C for 2 h, briefly rinsed three times in distilled water at room temperature, and dehydrated using a graded series of ethanol consisting of 30% (v/v), 50%, 70%, 80%, 90%, 100%, 100%, and 100% ethanol at 4°C for 10 min each. After the final change of 100% ethanol, the specimens were completely dried with the help of liquid CO_2_ using a critical point dryer (EM CPD300, Leica Microsystems), mounted on aluminum stubs using conductive carbon adhesive tapes, platinum-coated with a sputter coater (EM ACE200, Leica Microsystems), and finally examined by a field emission scanning electron microscope (Sigma, Carl Zeiss Microscopy GmbH) with an accelerating voltage of 5 kV and a working distance of 7.5 mm.

### Accession numbers

Sequence data from this article can be found in the Rice Genome Annotation Project (http://rice.uga.edu/analyses_search_locus.shtml), GenBank (https://www.ncbi.nlm.nih.gov/genbank/), and EMBL’s European Bioinformatics Institute (EMBL-EBI; https://www.ebi.ac.uk/) databases under accession numbers listed in **Supplementary Table S3**.

## Results

### OsMYB7 is a nucleus-localized R2R3-type MYB transcriptional repressor

**Figure 1:**
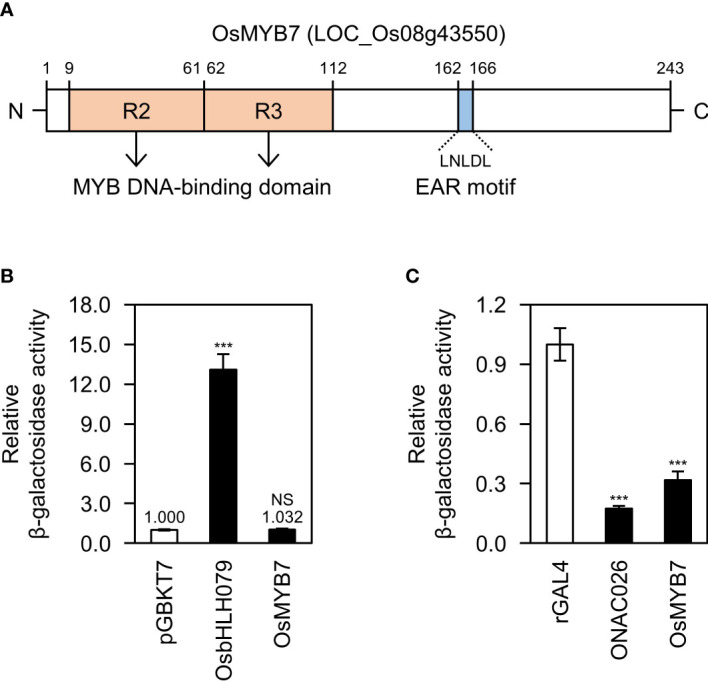
Biochemical characterization of OsMYB7. **(A)** Schematic diagram of the OsMYB7 protein. The two pale orange boxes indicate the highly conserved R2 and R3 MYB repeats. OsMYB7 also harbors an LxLxL-type EAR transcriptional repression motif, shown by the blue box. EAR, ethylene-responsive element binding factor-associated amphiphilic repression. **(B)** Transactivation activity assay of OsMYB7 in yeast. The full-length coding sequence of *OsMYB7* cloned in the vector pGBKT7 was subjected to a quantitative β-galactosidase liquid assay: *OsbHLH079* was used as a positive control, and pGBKT7 was used as a negative control. The β-galactosidase activity of each sample was normalized relative to that of the negative control, which was set to 1. Data are presented as means ± standard deviation (SD) from four independent yeast colonies. Significant differences between means were determined using two-tailed Student’s *t*-test (***P < 0.001). This experiment was performed twice with similar results. NS, not significant. **(C)** Trans-repression activity assay of OsMYB7 in yeast. The full-length coding sequence of *OsMYB7* cloned in the vector rGAL4 was subjected to a quantitative β-galactosidase liquid assay: *ONAC026* was used as a positive control, and rGAL4 was used as a negative control. The β-galactosidase activity of each sample was normalized to that of the negative control, which was set to 1. Data are presented as means ± SD of four independent yeast colonies. Asterisks indicate significant differences as determined by two-tailed Student’s *t*-test (***P < 0.001). This experiment was conducted twice yielding similar results. NS, not significant.

OsMYB7 is an R2R3-type MYB transcription factor in the MYB4/7/32 clade (Miyamoto et al., 2020) (**Figure 1A**). The MYB transcription factors in this clade, such as OsMYB108 from *Oryza sativa* (Miyamoto et al., 2019), ZmMYB42, ZmMYB31 from *Zea mays* (Sonbol et al., 2009; Fornalé et al., 2010), as well as AtMYB4, AtMYB7, and AtMYB32 from *Arabidopsis thaliana* (Jin et al., 2000; Preston et al., 2004; Fornalé et al., 2014), have been demonstrated to act as negative regulators of lignin biosynthesis. Multiple sequence alignment revealed that the OsMYB7 homologs contain a conserved LxLxL-type of ERF-associated Amphiphilic Repression (EAR) motif, a transcriptional repression motif (Kagale and Rozwadowski, 2011), along with a typical MYB DNA-binding domain composed of two R2 and R3 repeat motifs (Katiyar et al., 2012) (**Supplementary Figure S1**). In addition, we established that OsMYB7 exhibits strong trans-repression activity with no transactivating property in yeast (*Saccharomyces cerevisiae*) (**Figures 1B, C**): OsbHLH079 (Seo et al., 2020), a bHLH transcription factor with transactivation activity, was used as the positive control for transactivation assay, and ONAC026 (Mathew et al., 2016), a NAC transcription factor with trans-repression activity, was used as the positive control for trans-repression assay (see **Supplementary Table S3**). The results suggest that OsMYB7 acts as a transcriptional repressor in lignin biosynthesis.

OsMYB7 and its homologs are predicted to localize to the nucleus *in silico* (**Supplementary Table S1**). To clarify the subcellular location of OsMYB7 *in vivo*, we transiently expressed a construct encoding OsMYB7 fused to yellow fluorescent protein (*YFP-OsMYB7*) in onion epidermal cells. We detected YFP signals exclusively in the nucleus, confirming that OsMYB7 is a nucleus-localized protein (**Supplementary Figure S2**).

### OsMYB7 promotes leaf inclination at late stages of lamina joint development

To elucidate the physiological function of OsMYB7 in plant development, we obtained two different *OsMYB7* overexpressing lines (*OsMYB7-OE1* and *OsMYB7-OE2*) and an *osmyb7* knockout mutant (*osmyb7-1*) (**Supplementary Figure S3**). The *OsMYB7-OE1* line carries an activation-tagged T-DNA insertion at 80 nucleotides upstream of the start codon of *OsMYB7*, while the *OsMYB7-OE2* line contains a *35S::OsMYB7* transgene (**Supplementary Figures S3A–C**). RT-qPCR analysis confirmed high levels of *OsMYB7* transcripts in both overexpressing lines compared to the WT (**Supplementary Figure S3D**). The *osmyb7-1* mutant, generated using CRISPR/Cas9-mediated targeted mutagenesis, harbors a homozygous cytosine insertion in the second exon of *OsMYB7*, resulting in a truncated protein of 178 amino acids with a disrupted EAR motif and a portion of the DNA binding domain (**Supplementary Figures S3E–G**).

**Figure 2:**
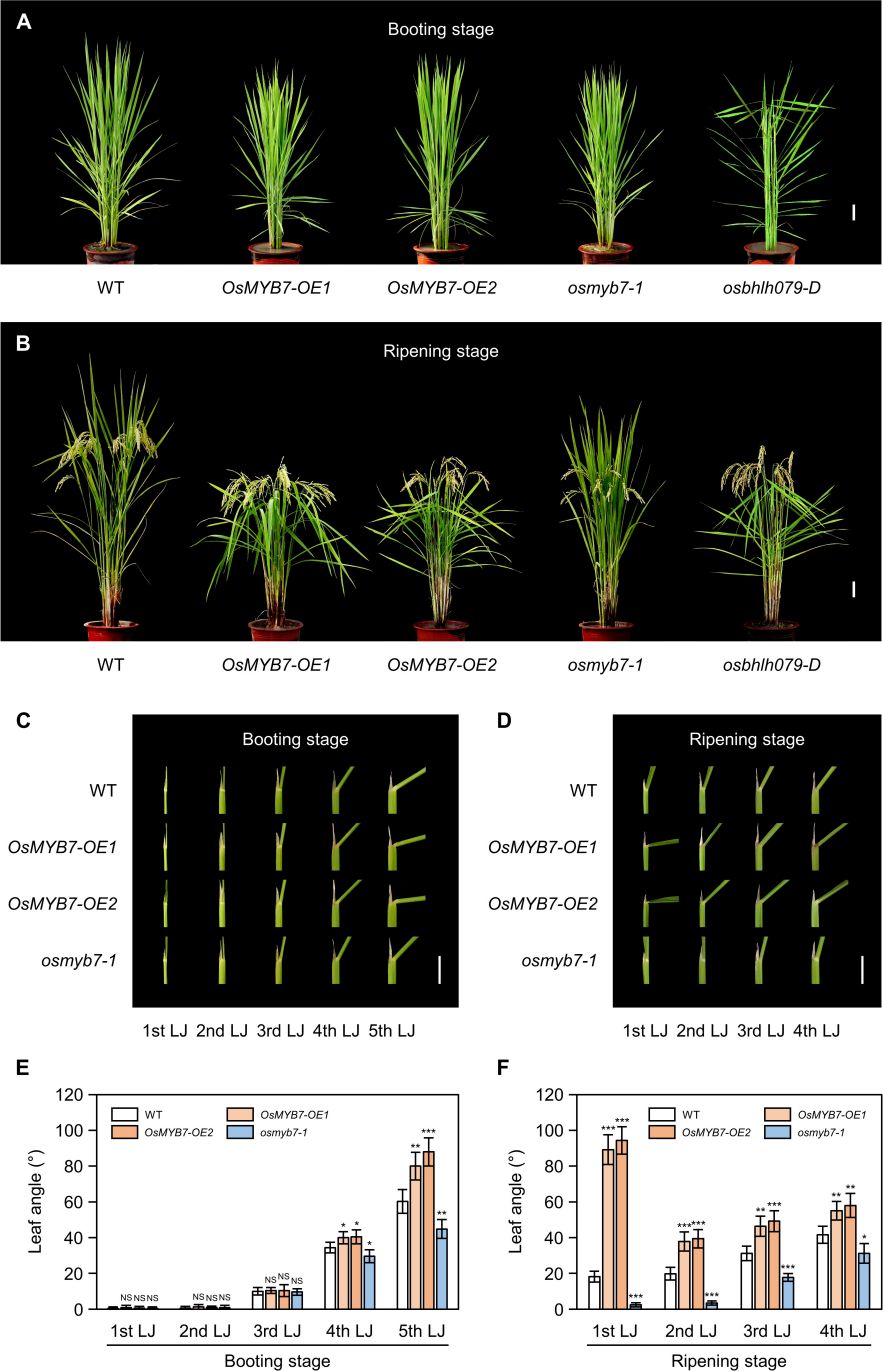
OsMYB7 determines leaf angle at the late stage of lamina joint development. **(A, B)** Phenotypes of WT, *OsMYB7-OE1*, *OsMYB7-OE2*, *osmyb7-1*, and *osbhlh079-D* plants at the booting stage (105 DAS) **(A)** and at the ripening stage (30 DAH) **(B)** grown under natural long day conditions in a paddy field. The *osbhlh079-D* plant represents a typical mutant with increased leaf angles due to enhanced BR signaling. Scale: 10 cm. **(C, D)** Lamina joints of WT, *OsMYB7-OE1*, *OsMYB7-OE2*, and *osmyb7-1* plants. Samples in **(C, D)** were obtained from the plants shown in **(A,B)**, respectively. Scale: 1 cm. **(E, F)** Leaf angle in WT, *OsMYB7-OE1*, *OsMYB7-OE2*, and *osmyb7-1* plants at the booting stage (105 DAS) **(E)** and at the ripening stage (30 DAH) **(F)** grown in a paddy field. Data are presented as means ± SD from five independent plants (5 leaf angles per plant). Asterisks indicate significant differences compared to WT as determined by two-tailed Student’s *t*-test (*P < 0.05, **P < 0.01, and ***P < 0.001). DAH, days after heading; DAS, days after sowing; NS, not significant; *n*th LJ, lamina joint of *n*th leaf.

We observed altered leaf angle phenotypes in these lines under natural long-day conditions in a paddy field. At the booting stage (105 days after sowing [DAS]), we detected significant changes in leaf angles in the 4th and 5th leaves: *OsMYB7* overexpressors showed wide leaf angles, whereas the *osmyb7-1* mutant had narrow leaf angles compared to the parental *japonica* rice cultivar ‘Donglin’ (hereafter termed wild type; WT) (**Figure 2A**). The differences in leaf angles of the 1st, 2nd, and 3rd leaves, also began to emerge at around 10 days after heading (DAH) and became more pronounced at the grain ripening stage (30 DAH) (**Figure 2B**): all leaves of *OsMYB7* overexpressors took on a more horizontal orientation, while those of the *osmyb7-1* mutant were in a more vertical orientation compared to WT. Consistent with the field observations, only the 4th and 5th leaf angles showed differences at the booting stage among the examined plants (**Figures 2C, E**), although the leaf angles of all leaves in *OsMYB7* overexpressors and the *osmyb7-1* mutant were markedly altered at the ripening stage (**Figures 2D, F**).

It is worth noting that the phenotypes in *OsMYB7* overexpressors and the *osmyb7-1* mutant were somewhat distinct from those reported in other rice mutants with altered leaf inclinations, such as *osbhlh079-D* (Seo et al., 2020), *OsWRKY53-OE* (Tian et al., 2017), and *lc2-1* (Zhao et al., 2010), in that the phenotype was restricted to the lamina joints at the late developmental stage. For example, *osbhlh079-D*, a typical mutant with enhanced BR signaling, showed enlarged leaf angles throughout its life cycle, including at the booting stage (**Figures 2A, B**). Collectively, these observations indicate that OsMYB7 plays a role in enlarging leaf angles in a developmental stage-specific manner.

### *OsMYB7* is preferentially expressed in lamina joints at S5 developmental stage

**Figure 3:**
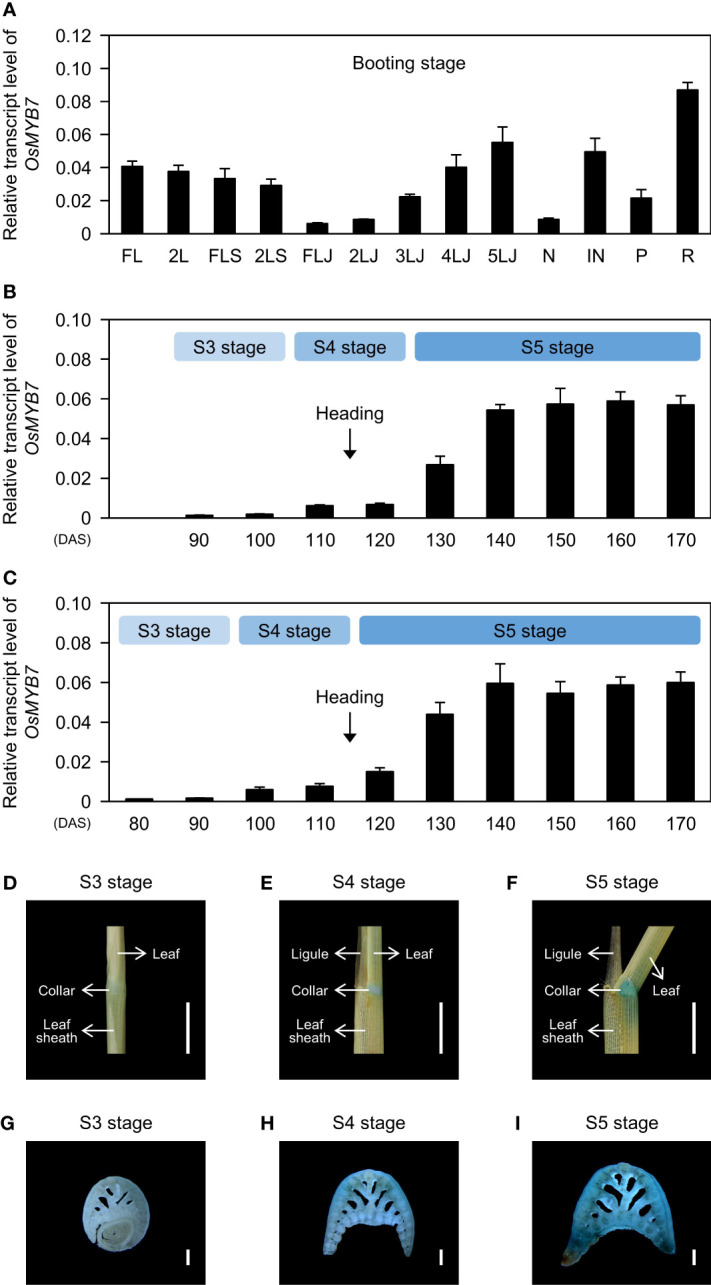
Expression profiles of *OsMYB7*. **(A)** Spatial expression patterns of *OsMYB7* across tissues. Each tissue designated in **(A)** was collected from WT at the booting stage grown under natural day-night conditions in a paddy field. The transcript levels of *OsMYB7* determined by RT-qPCR analysis were normalized to those of *GAPDH* and presented as means ± SD from four biological replicates. FL, flag leaf; 2L, 2nd leaf; FLS, flag leaf sheath; 2LS, 2nd leaf sheath; FLJ, lamina joint of flag leaf; *n*LJ, lamina joint of *n*th leaf; N, node; IN, internode; P, panicle; R, root. **(B, C)** Temporal expression patterns of *OsMYB7* in the flag leaf lamina joint **(B)** and the 2nd leaf lamina joint **(C)** across developmental stages. Each lamina joint was collected every 10 days between S3 and S5 developmental stages from WT grown under natural paddy field conditions. The expression levels of *OsMYB7* determined by RT-qPCR analysis were normalized to those of *GAPDH* and presented as means ± SD from four biological samples (around 30 lamina joints per sample). Each developmental stage is indicated in the light blue boxes. The average time of heading in WT is shown by black arrows. DAS, days after sowing. **(D-I)** Histochemical analysis of GUS activity derived from the *OsMYB7* promoter. GUS activity in the 2nd leaf lamina joint at S3 **(D)**, S4 **(E)**, and S5 **(F)** stage, and the corresponding transverse section of the 2nd leaf lamina joint at S3 **(G)**, S4 **(H)**, and S5 **(I)** stages. Samples were collected from *OsMYB7-OE1*, which harbors a T-DNA with a promoterless *β-glucuronidase* (*GUS*) reporter at 80 bp upstream of the ATG initiation site in *OsMYB7*, grown under natural long-day conditions. A schematic diagram illustrating the position of the T-DNA insertion in *OsMYB7-OE1* is shown in **Supplementary Figure S3A**. Scales: 5 mm **(D-F)** and 500 μm **(G–I)**. Similar results were repeated in at least three independent samples.

The spatiotemporal pattern of gene expression holds fundamental information for understanding the physiological functions of the encoded protein. Therefore, we determined the tissue specificity of the expression of *OsMYB7*. Interestingly, *OsMYB7* exhibited a gradual increase in expression from the flag lamina joints to the 5th lamina joints (**Figure 3A**), leading us to hypothesize that *OsMYB7* transcript levels in the lamina joints are developmentally regulated. To test this hypothesis, we monitored changes in *OsMYB7* expression at the flag- and 2nd-leaf lamina joints across developmental age. Even though the development of lamina joints is divided into six successive stages from initiation (S1) to senescence (S6) based on their morphological features (Zhou et al., 2017) (**Supplementary Table S2**), we focused on the S3, S4, and S5 stages for simplicity: the lamina joint is encircled by the prior leaf sheath at the S3 stage, and is exposed to the air at the S4 stage, before bending at the S5 stage. Reverse-transcription quantitative PCR (RT-qPCR) analysis revealed that *OsMYB7* expression levels gradually increase throughout development, with the sharpest rise at the onset of the S5 stage (**Figures 3B, C**). Furthermore, subsequent histochemical analysis of GUS (ß-glucuronidase) activity derived from *GUS* expression driven by the *OsMYB7* promoter revealed that the collar at S5 stage exhibited intense GUS staining, while that at S3 stage showed faint *GUS* expression (**Figures 3D–I**). These results support that the transcription levels of *OsMYB7* in the lamina joints are tightly controlled during development and preferentially upregulated at the S5 stage of lamina joints, in agreement with the phenotypic observations (**Figure 2**).

### OsMYB7 acts as a pathway-wide transcriptional repressor of lignin biosynthesis

**Figure 4:**
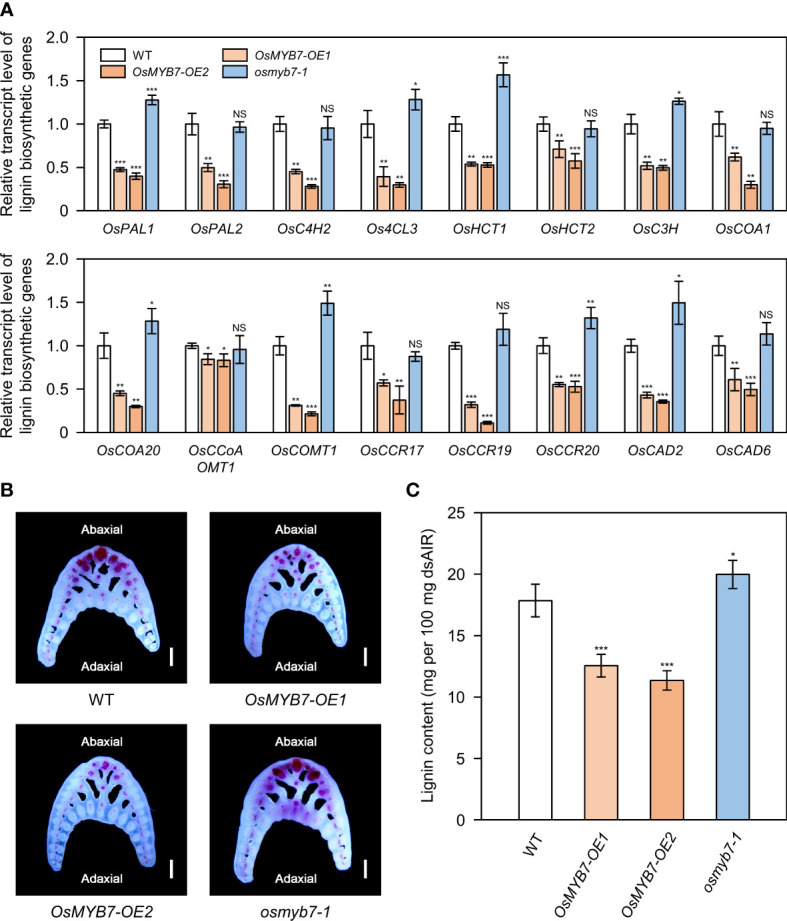
OsMYB7 negatively regulates lignin biosynthesis at the lamina joint. **(A)** Relative transcript levels of lignin biosynthetic genes in WT, *OsMYB7-OE1*, *OsMYB7-OE2*, and *osmyb7-1* plants. Samples were harvested from the flag leaf lamina joints at the S5 stage from plants grown under natural long-day conditions in a paddy field. The expression levels of each gene determined by RT-qPCR analysis were normalized to those of *GAPDH* and represented relative to WT, which was set to 1. Data are presented as means ± SD from four biological samples (around 30 lamina joints per sample). Asterisks indicate significant differences as determined by two-tailed Student’s *t*-test (*P < 0.05, **P < 0.01, and ***P < 0.001). These experiments were conducted twice with similar results. NS, not significant. **(B)** Histochemical staining of the lamina joint for lignin. Flag leaf lamina joints of WT, *OsMYB7-OE1*, *OsMYB7-OE2*, and *osmyb7-1* plants at the ripening stage (30 DAH) grown in a natural paddy field were collected, and the corresponding transverse hand sections were subjected to phloroglucinol-HCl staining, which visualizes lignin as a pink to red color. Scale: 500 μm. Similar results were obtained in at least three independent samples. **(C)** Determination of lignin contents in lamina joints of WT, *OsMYB7-OE1*, *OsMYB7-OE2*, and *osmyb7-1* plants. Lamina joints of flag leaves at the S5 developmental stage were collected from plants grown under natural long-day conditions and subjected to acetyl bromide assay, which quantifies total lignin contents. Data are presented as means ± SD from six independent samples (around 50 lamina joints per sample); asterisks indicated significant differences as determined by two-tailed Student’s *t*-test (*P < 0.05 and ***P < 0.001). These experiments were repeated twice, yielding similar results. dsAIR, destarched alcohol-insoluble cell wall residue.

Lignin modulates leaf angle by determining the mechanical strength of lamina joints (Sun et al., 2020; Wang R. et al., 2020). Thus, we speculated that OsMYB7 might dictate leaf angle by controlling lignin biosynthesis. To test whether OsMYB7 function is closely associated with lignin, we examined the transcript levels of 16 lignin biosynthetic genes (see **Supplementary Table S3**). We determined that transcript abundances of these lignin biosynthetic genes were significantly altered in *OsMYB7* overexpressors and the *osmyb7-1* mutant in opposite directions: they were downregulated in *OsMYB7* overexpressors and upregulated in the *osmyb7-1* mutant (**Figure 4A**).

We also conducted histochemical staining of lignin from lamina joint cross-sections. Consistent with previous reports (Wang R. et al., 2020; Huang et al., 2021), lignin deposition was mainly observed in sclerenchyma cells and vascular bundles on the abaxial side of lamina joints (**Figure 4B**). We detected weaker lignin signals in the abaxial sclerenchyma cells of *OsMYB7-OE1* and *OsMYB7-OE2* lines compared to the WT, while the sclerenchyma cells on the adaxial side were not lignin-rich in both WT and *OsMYB7* overexpressors (**Figure 4B**). In contrast, the functional deficiency of *OsMYB7* resulted in noticeable ectopic lignification in the adaxial sclerenchyma cells, although no significant difference in lignin deposition was observed on the abaxial tissues between WT and *osmyb7-1*, possibly because the lignin on the abaxial side was fully saturated in the WT (**Figure 4B**). In support of these observations, lignin contents in the lamina joints were 30~36% lower in *OsMYB7* overexpressors and 12% higher in the *osmyb7-1* mutant, compared to those in WT (**Figure 4C**). These results thus confirm that OsMYB7 has a negative role in lignin biosynthesis at the lamina joint.

### OsMYB7 activates a biosynthetic program for cellulose at lamina joints

**Figure 5:**
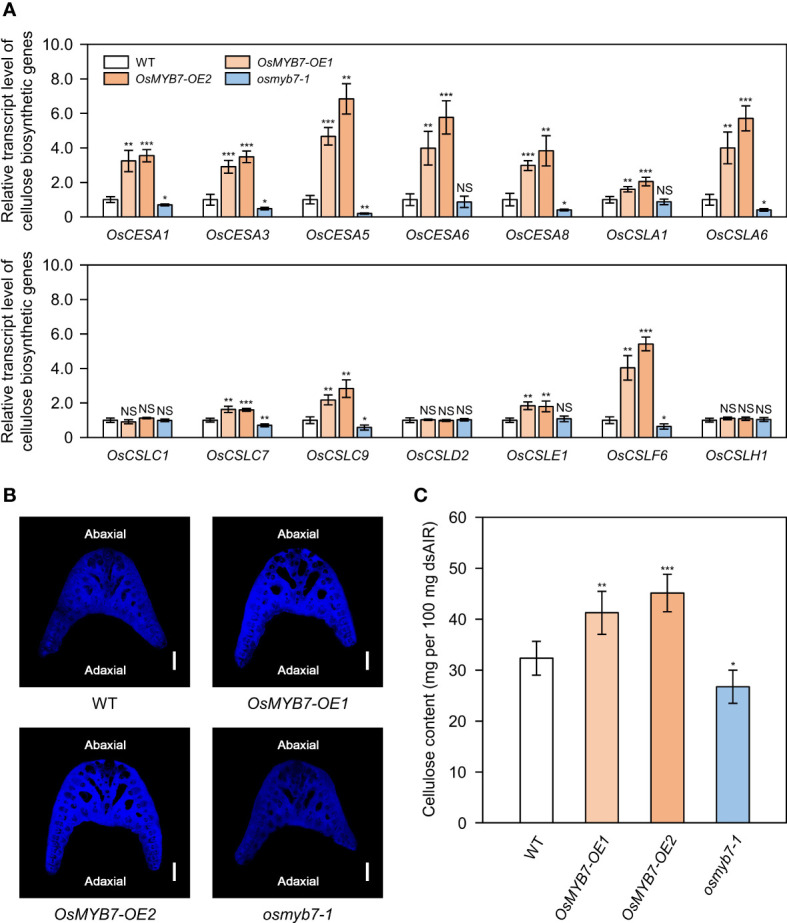
OsMYB7 is a positive regulator of cellulose biosynthesis at the lamina joint. **(A)** Relative transcript levels of cellulose biosynthetic genes in WT, *OsMYB7-OE1*, *OsMYB7-OE2*, and *osmyb7-1* plants. Samples obtained in **Figure 4A** were subjected to RT-qPCR analysis. The expression levels of each gene were normalized to those of *GAPDH* and presented relative to the WT, which was set to 1. Data are presented as means ± SD. Asterisks indicate significant differences as determined by two-tailed Student’s *t*-test (*P < 0.05, **P < 0.01, and ***P < 0.001). These experiments were conducted twice yielding similar results. NS, not significant. **(B)** Histochemical staining of the lamina joint for cellulose. Lamina joints of flag leaves at the S5 stage were collected from WT, *OsMYB7-OE1*, *OsMYB7-OE2*, and *osmyb7-1* plants grown in natural paddy field conditions, and the corresponding transverse sections were subjected to calcofluor white staining, which visualizes cellulose as bright blue color under UV excitation. Scale: 500 μm. Similar results were obtained in at least three independent samples. **(C)** Determination of cellulose contents in lamina joints of WT, *OsMYB7-OE1*, *OsMYB7-OE2*, and *osmyb7-1* plants. Flag leaf lamina joints from plants at the ripening stage (30 DAH) grown under natural day-night conditions in a paddy field were harvested and subjected to anthrone assay, which quantifies total cellulose contents colorimetrically. Data are presented as means ± SD from six independent samples (around 50 lamina joints per sample). Asterisks indicate significant differences as determined by two-tailed Student’s *t*-test (*P < 0.05, **P < 0.01, and ***P < 0.001). These experiments were repeated twice with similar results. dsAIR, destarched alcohol-insoluble cell wall residue.

Cellulose, together with lignin, controls leaf inclination by affecting the mechanical properties of sclerenchymatous tissues in the lamina joints (Ning et al., 2011; Sun et al., 2020; Huang et al., 2021). In addition, some rice MYB transcriptional activators (OsMYB46, OsMYB58/63, and OsMYB103) of the lignin biosynthetic pathway trigger cellulose biosynthesis (Zhong et al., 2011; Hirano et al., 2013; Noda et al., 2015), leading us to speculate that OsMYB7 may coordinately repress the biosynthesis of SCW components. To assess whether OsMYB7 is involved in cellulose accumulation, we examined the expression levels of 14 genes from the *CELLULOSE SYNTHASE/CELLULOSE SYNTHASE-LIKE* (*OsCESA*/*CSL*) superfamily (see **Supplementary Table S3**), all of which are expressed abundantly in the lamina joints at the S5 stage (Zhou et al., 2017). Among them, 11 genes showed elevated expression in *OsMYB7* overexpressors, and at least 8 genes exhibited decreased expression in the *osmyb7-1* mutant, compared to WT (**Figure 5A**). Moreover, cellulose staining on the transverse sections of lamina joints revealed that *OsMYB7* overexpressors and the *osmyb7-1* mutant tend to show stronger and weaker signals, respectively, than WT (**Figure 5B**). We confirmed these observations by anthrone assays, which quantify the contents of total crystalline cellulose; cellulose was overproduced and underproduced in the lamina joints of *OsMYB7* overexpressors and the *osmyb7-1* mutant, respectively (**Figure 5C**). Taken together, OsMYB7 promotes cellulose deposition at the lamina joints at the S5 stage.

### SCW thickening is accelerated by OsMYB7

The thickness of the SCW is determined by the amount of deposited lignin and cellulose and affects leaf angle as a primary decisive factor of mechanical strength (Sun et al., 2020; Huang et al., 2021). However, it was difficult to simply assume that OsMYB7 inhibits thickening of SCW by reducing lignin accumulation, because it promoted deposition of cellulose (**Figures 4**, **5**). To pinpoint the difference in SCW thickness, we examined sclerenchyma cells in the lamina joints using a transmission electron microscope. Interestingly, compared to WT, *OsMYB7* overexpressors had moderately thicker SCWs, whereas the *osmyb7-1* mutant showed a slight reduction in SCW thickness (**Supplementary Figure S4**). These results suggest that the inhibition of lignin deposition by OsMYB7 may not be the main cause of alteration in leaf angle. However, we cannot exclude the possibility that other phenylpropanoids, in addition to lignin, may also influence the leaf angle phenotype.

### OsMYB7-mediated regulation of leaf angle is independent of BRs

Leaf angle has been widely considered to be determined by the balance between two opposing forces: pushing and supporting forces. The pushing force is primarily derived from preferential cell division/elongation at the adaxial side of lamina joints. The supporting force is based on abundant sclerenchymatous cells with thick SCW in the lamina joints (Wang R. et al., 2020; Xu et al., 2021). Therefore, we focused on the pushing force, which is mainly established under phytohormonal control (Xu et al., 2021). To elucidate whether OsMYB7-mediated leaf angle formation is orchestrated by phytohormones, we examined the transcript levels of representative BR biosynthetic and signaling genes (see **Supplementary Table S3**), because BRs play a dominant role in adaxial cell division/elongation at the lamina joints (Li et al., 2020). The expression levels of BR-related genes in *OsMYB7* overexpressors and the *osmyb7-1* mutant were almost identical to those of WT (**Supplementary Figure S5**). Furthermore, a BR-induced lamina joint inclination assay (Wada et al., 1984) confirmed that the sensitivity of *OsMYB7* overexpressors and the *osmyb7-1* mutant are not altered by BR treatments, compared to the WT (**Supplementary Figure S6**). Taken together, these findings suggest that OsMYB7 is involved in the regulation of leaf angles in a BR-independent manner.

### Free auxin levels at lamina joints were altered in *OsMYB7* overexpressors and *osmyb7-1* mutant

**Figure 6:**
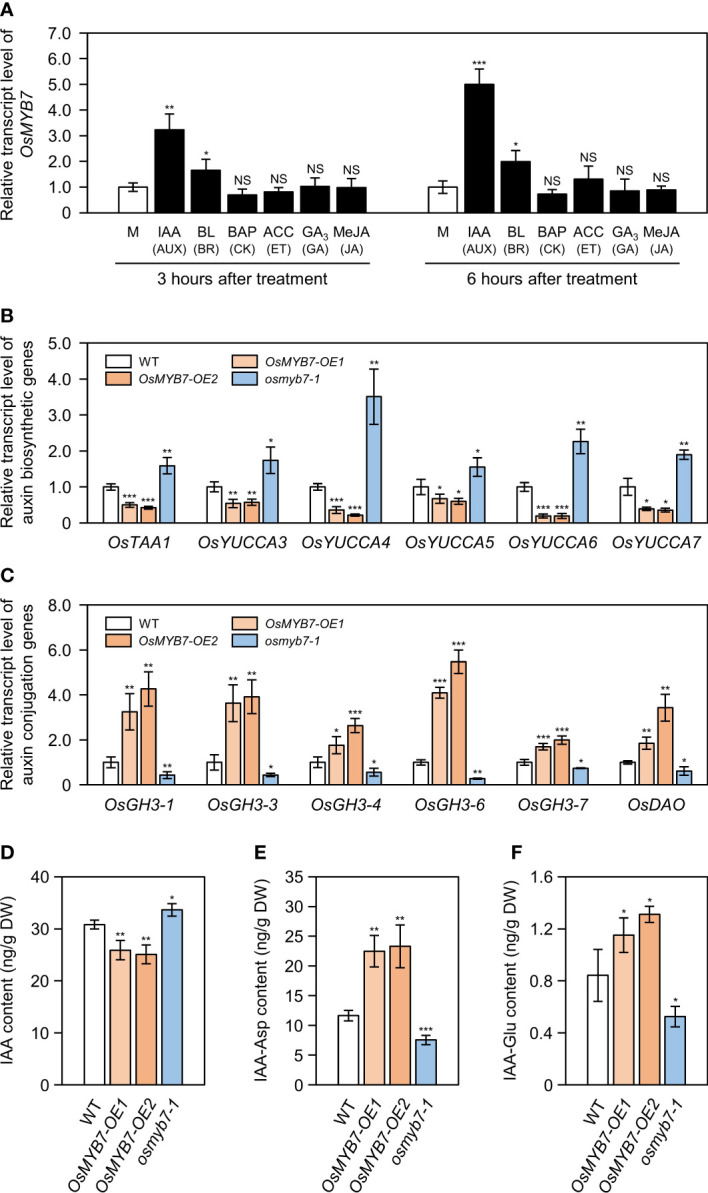
Alteration in free auxin levels at lamina joints may be one cause of different leaf angle phenotypes among WT, *OsMYB7-OE1*, *OsMYB7-OE2*, and *osmyb7-1*. **(A)** Relative *OsMYB7* expression levels in response to various phytohormones determining leaf angle. WT seedlings were grown on half-strength solid MS medium for ten days under constant light conditions at 28°C, followed by incubation in half-strength liquid MS medium containing with 100 μM IAA, 10 μM BL, 10 μM BAP, 10 mM ACC, 100 μM GA_3_, or 100 μM MeJA. WT seedlings incubated in half-strength liquid MS medium without phytohormone were used as a mock. Total RNA was extracted from shoots collected after 3 or 6 h of treatment, and used for RT-qPCR analysis. The transcript levels of *OsMYB7* were normalized to those of *GAPDH*, with the expression level in the phytohormone-treated group shown relative to that in the mock-treated group, which was set to 1. Data are presented as means ± SD from four biological samples (around 3 shoots per sample). Asterisks indicate significant differences compared to the mock-treated sample, as determined by two-tailed Student’s *t*-test (*P < 0.05, **P < 0.01, and ***P < 0.001). This experiment was performed independently twice and similar results were obtained. AUX, auxin; BR, brassinosteroid; CK, cytokinin; ET, ethylene; GA, gibberellin; JA, jasmonic acid; M, mock; NS, not significant. **(B, C)** Relative expression of auxin biosynthetic **(B)** and conjugation **(C)** genes at the lamina joint. The cDNA samples in **Figure 4A** were subjected to RT-qPCR analysis. *GAPDH* was used as an internal control and the relative expression of each gene is shown relative to that in WT (set to 1). Data are presented as means ± SD of four biological replicates. Asterisks indicate significant differences as determined by two-tailed Student’s *t*-test (*P < 0.05, **P < 0.01, and ***P < 0.001). These experiments were conducted twice yielding similar results. **(D–F)** Contents for free IAA and IAA-amino acid conjugates (IAA-Asp and IAA-Glu). Flag leaf lamina joints at the S5 developmental stage in WT, *OsMYB7-OE1*, *OsMYB7-OE2*, and *osmyb7-1* plants were collected and subjected to LC-ESI-MS/MS analysis for quantification of free IAA **(D)**, IAA-Asp **(E)**, and IAA-Glu **(F)**. Data are presented as means ± SD from four biological replicates (around 10 lamina joints per plant and around 15 plants per replicates). Asterisks indicate significant differences as determined by two-tailed Student’s *t*-test (*P < 0.05, **P < 0.01, and ***P < 0.001). DW, dry weight; IAA, indole-3-acetic acid; IAA-Asp, IAA-aspartate; IAA-Glu, IAA-glutamate.

Besides BRs, other phytohormones, including auxin, cytokinin, ethylene, gibberellin, and jasmonate, comprehensively participate in controlling leaf inclination (Li et al., 2020; Xu et al., 2021). Accordingly, we investigated whether other phytohormones might affect the expression of *OsMYB7* by RT-qPCR. Although *OsMYB7* expression was slightly induced in response to 24-epibrassinolide (BL; the active form of BR), it was strikingly upregulated 3.24 and 5.00 times after three and six hours of treatment with auxin (indole-3-acetic acid, IAA), respectively, compared to that in the mock samples (**Figure 6A**). In support of these results, we identified ten putative auxin-responsive elements (AuxREs), including two canonical AuxREs (Mironova et al., 2014; Lanctot et al., 2020), in the promoter region of *OsMYB7* (−2,000 bp to −1 bp from the start codon) (**Supplementary Figure S7**), suggesting that OsMYB7 is associated with auxin-dependent lamina inclination.

To evaluate whether the leaf angle phenotypes of *OsMYB7* overexpressors and the *osmyb7-1* mutant were associated with endogenous IAA contents, we measured the expression levels of auxin biosynthetic genes and auxin conjugation genes in the lamina joints (see **Supplementary Table S3**). We determined that auxin anabolic genes (*OsTAA1*, *OsYUCCA3*, *OsYUCCA4*, *OsYUCCA5*, *OsYUCCA6*, and *OsYUCCA7*) are severely downregulated in *OsMYB7* overexpressors and upregulated in the *osmyb7-1* mutant (**Figure 6B**). By contrast, auxin conjugation genes (*OsGH3-1*, *OsGH3-3*, *OsGH3-4*, *OsGH3-6*, *OsGH3-7*, and *OsDAO*) were more highly expressed in *OsMYB7* overexpressors and less so in the *osmyb7-1* mutant compared to the WT (**Figure 6C**). Consistent with the transcript levels of auxin biosynthetic genes, endogenous free IAA levels in the lamina joints were approximately 1.2-fold lower in *OsMYB7* overexpressors and 1.09-fold higher in the *osmyb7-1* mutant than the WT (**Figure 6D**), whereas IAA conjugates, IAA-Asp and IAA-Glu, were more concentrated in the lamina joints of *OsMYB7* overexpressors and less concentrated in those of the *osmyb7-1* mutant (**Figures 6E, F**). All of these findings suggest that OsMYB7 may promote rice lamina inclination by inhibiting the accumulation of free auxin in lamina joints.

### OsMYB7 affects cell elongation at the adaxial side of the lamina joint

**Figure 7:**
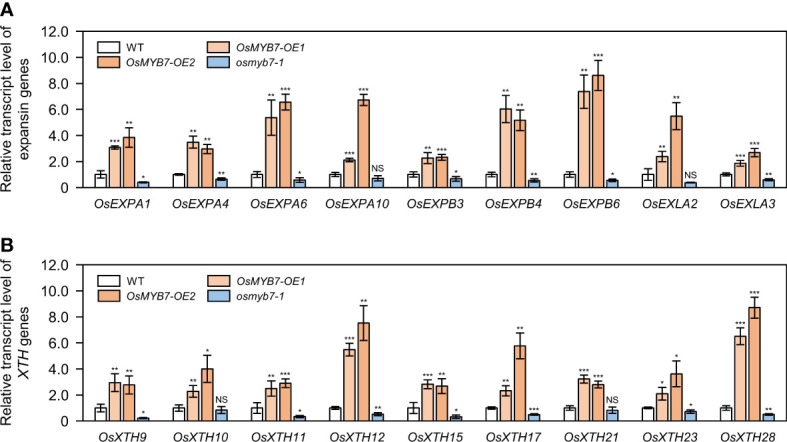
Expression analysis of cell elongation-related genes at the lamina joint. **(A, B)** Relative transcript levels of expansin **(A)** and *XTH* **(B)** genes in WT, *OsMYB7-OE1*, *OsMYB7-OE2*, and *osmyb7-1* plants. Total RNA samples obtained in **Figure 4A** were used for RT-qPCR analysis. The expression level of each examined gene was normalized to that of *GAPDH*, and shown relative to WT levels, which were set to 1. Data are presented as means ± SD (*n* = 4); asterisks indicate significant differences as determined by two-tailed Student’s *t*-test (*P < 0.05, **P < 0.01, and ***P < 0.001). These experiments were carried out twice with independent biological replicates, and similar results were obtained. NS, not significant; XTH, xyloglucan endotransglucosylase/hydrolase.

Auxin deficiency boosts parenchymal cell division and/or elongation at the adaxial side of lamina joints, resulting in enlarged leaf angles (Zhao et al., 2013; Zhang et al., 2015). To unravel the cellular mechanism of OsMYB7 in leaf inclination, we investigated the effect of OsMYB7 on the expression of cell division- and cell elongation-associated genes at the lamina joints. The transcript levels of cell division-related genes, such as cyclin and CDK genes (see **Supplementary Table S3**), were not altered in *OsMYB7* overexpressors or the *osmyb7-1* mutant (**Supplementary Figure S8**). By contrast, we observed significant changes in expression of cell elongation-related genes, such as *EXPANSIN* and *XYLOGLUCAN ENDOTRANSGLUCOSYLASE*/*HYDROLASE* (*XTH*) genes (see **Supplementary Table S3**), in *OsMYB7* overexpressors and the *osmyb7-1* mutant; these genes were upregulated in *OsMYB7* overexpressors and downregulated in the *osmyb7-1* mutant (**Figure 7**). This observation indicated that OsMYB7 may play a positive role in the promotion of lamina inclination by stimulating adaxial cell elongation rather than cell division.

**Figure 8:**
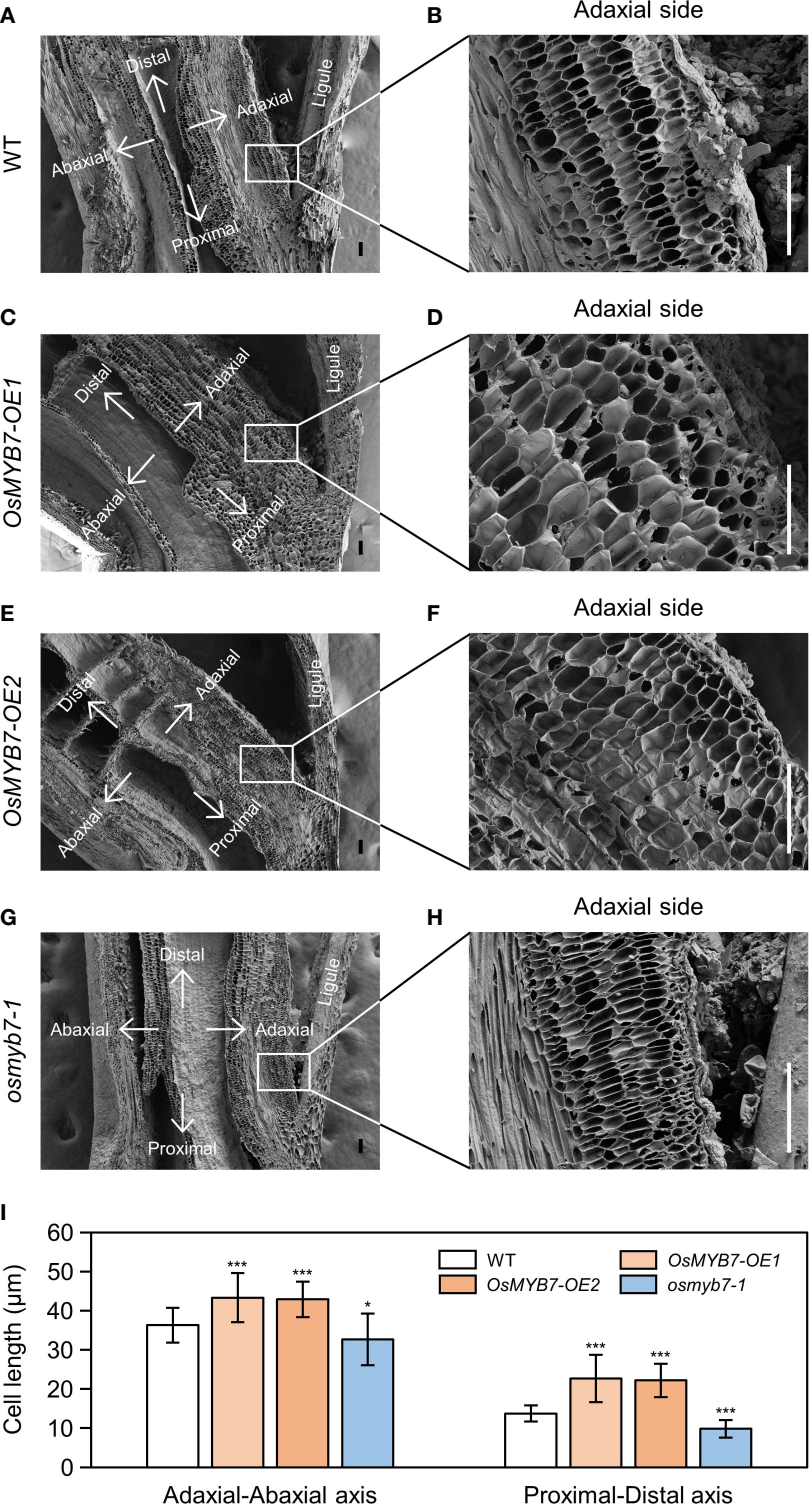
Scanning electron microscopy examination of the lamina joint. **(A–H)** Longitudinal sections of flag leaf lamina joints at the S5 developmental stage in WT **(A)**, *OsMYB7-OE1* **(C)**, *OsMYB7-OE2* **(E)**, and *osmyb7-1* **(G)** plants grown under natural long-day conditions in a paddy field. Close-up views of the adaxial region denoted by rectangles in **(A**, **C**, **E, G)** are shown in **(B**, **D**, **F**, **H)**, respectively. Scale: 100 μm. **(I)** Adaxial cell length in **(B**, **D**, **F**, **H)**. Length of the adaxial cells was measured along the adaxial-abaxial axis and proximal-distal axis. Data are presented as means ± SD from 30 cells. Asterisks indicate significant differences compared to WT, as determined by two-tailed Student’s *t*-test (*P < 0.05 and ***P < 0.001). Similar results were obtained from at least three independent samples.

For further verification, we examined longitudinal sections of lamina joints using a scanning electron microscope. In agreement with the gene expression profiles, the adaxial cells in *OsMYB7* overexpressors were elongated both longitudinally and transversely to a greater extent than the WT, while those in the *osmyb7-1* mutant failed to expand (**Figure 8**). This result demonstrates that OsMYB7-mediated leaf angle formation at least partially depends on parenchymal cell size at the adaxial side of lamina joints.

## Discussion

### Lignin and cellulose depositions at the lamina joints appear to be regulated under a compensatory balance mechanism

Several cell wall-associated MYB transcription factors regulate lignin accumulation in rice, most of which function as pathway-wide transcriptional activators of lignin biosynthesis (Miyamoto et al., 2020). The rice MYB activators OsMYB46, OsMYB58/63, OsMYB61a, and OsMYB103L have also have been shown to promote cellulose biosynthesis (Zhong et al., 2011; Yang et al., 2014; Noda et al., 2015; Zhao et al., 2019). In Arabidopsis, *OsMYB46* overexpression induced ectopic deposition of secondary cell wall components (lignin, cellulose, and xylan) in the walls of the epidermis (Zhong et al., 2011). A knockdown mutation in *OsMYB103L* decreased lignin and cellulose accumulation, which resulted in brittle leaves (Hirano et al., 2013; Yang et al., 2014). Moreover, there is some evidence that these types of MYB transcription factors directly induce the expression of cellulose synthase genes (Noda et al., 2015). We thus postulated that OsMYB7 might be involved in suppressing the biosynthesis of both lignin and cellulose.

In this study, we demonstrated that OsMYB7 activated the cellulose biosynthetic program, although it inhibited lignin deposition (**Figures 4**, **5**). These findings raised the question as to whether OsMYB7 directly triggered the transcription of cellulose biosynthetic genes: importantly, OsMYB7 did not possess transactivation activity for the direct activation of downstream genes (**Figure 1B**). In the last few decades, lignin and cellulose deposition has been suggested to be regulated in a compensatory fashion in some plant species, such as rice, maize, and aspen (*Populus tremuloides*). For example, mutants lacking cellulose, including *brittle culm 7* (*bc7*(*t*)) in rice and *brittle stalk-2* (*bk2*) or *bk-5* in maize, exhibited a significant increase in their lignin contents, and transgenic aspens with lower transcript levels for *4-COUMARATE : COA LIGASE* (*Pt4CL1*), one of the lignin biosynthetic genes, showed a concomitant increase in cellulose (Hu et al., 1999; Sindhu et al., 2007; Wei et al., 2011; Li et al., 2022). Furthermore, in the case of transgenic rice plants overexpressing Arabidopsis *SHINE* (*SHN*), the compensatory increase in cellulose was shown to offset any defects in mechanical strength due to lack of lignin (Ambavaram et al., 2011). Conclusively, we suggest that the promotion of cellulose deposition by OsMYB7 may result from compensation for lignin deficiency.

### OsMYB7 promotes leaf inclination possibly through accelerating a decline in free auxin levels at the late stage of lamina joint development

MYB proteins comprise one of the largest transcription factor superfamilies in plants and play crucial roles in multiple biological functions, including plant development, phytohormonal signaling, secondary metabolism, and response to environmental stress (Jin and Martin, 1999; Dubos et al., 2010; Liu et al., 2015). Among the 155 putative/known MYB transcription factors in rice (Katiyar et al., 2012), a contribution to leaf angle has only been attributed to one MYB member, GIBBERELLIN-INDUCIBLE MYB-LIKE 2 (OsGAMYBL2), which is responsible for integrating BRs and gibberellin signaling pathways at lamina joints to maintain leaf erectness (Gao et al., 2018). In this study, we identified a new MYB transcription factor, OsMYB7, as influencing leaf inclination through modulating auxin homeostasis (**Figures 2**, **6**). Interestingly, OsMYB7 presented the specific characteristic that its phenotypic effects are restricted to lamina joints at the late developmental stage, coinciding with the findings that expression of *OsMYB7* in the lamina joints is drastically upregulated at the S5 stage (**Figures 2**, **3**). Recently, the concentration of active auxin, IAA, was revealed to gradually decline over lamina joint development (Zhou et al., 2017), although the underlying mechanism is largely unknown. Since auxin stimulates cell division and suppresses cell elongation when accumulating at high levels (Chen et al., 2001), it has been widely considered that auxin is responsible for rapid increase in cell number at the developing lamina joints (Zhou et al., 2017). However, auxin induces cell elongation when present at low levels (Chen et al., 2001), and reduced auxin contents in lamina joints at the S5 developmental stage give rise to excessive cell elongation at the adaxial side of lamina joints, promoting lamina joint bending (Zhao et al., 2013; Zhou et al., 2017). Combined with our data, the preferential expression of *OsMYB7* in the lamina joints at the late developmental stage may partially contribute to the incremental decrease in auxin contents over lamina joint development, thereby accelerating adaxial cell elongation and leaf inclination at the S5 stage.

### Perturbations of auxin homeostasis in *OsMYB7* overexpressors and the *osmyb7-1* mutant might result from changes in lignin quantity

Auxin acts upstream of the lignin biosynthetic pathway (Qu et al., 2021). Overexpression of auxin biosynthetic genes, such as *YUCCA8* and *YUCCA9*, led to strong lignification of aerial tissues in Arabidopsis (Hentrich et al., 2013). Although the effect of lignin pathway perturbations on auxin homeostasis has been less studied, there is some evidence that altering lignin levels can affect the expression of auxin-related genes. For instance, loss-of-function mutations of lignin biosynthetic genes, such as *CINNAMATE 4-HYDROXYLASE* (*C4H*), *4CL1*, *CAFFEOYL-COA O-METHYLTRANSFERASE 1* (*CCoAOMT1*), and *CINNAMOYL-COA REDUCTASE 1* (*CCR1*), in Arabidopsis caused a drop in the transcript levels of many auxin-responsive genes (Vanholme et al., 2012). Rogers et al. (2005) revealed that the auxin biosynthetic gene *CYTOCHROME P450 79B2* (*CYP79B2*) was upregulated in all investigated ectopic lignification mutants, including *de-etiolated 3* (*det3*), *pom-pom 1* (*pom1*), and *ectopic lignification 1* (*eli1*), suggesting feedback regulation between lignin and auxin biosynthesis in Arabidopsis. Based on our results, we propose that the alterations in lignin deposition at the lamina joints of both *OsMYB7* overexpressors and the *osmyb7-1* mutant may contribute to changes in auxin levels. Similarly, low-lignin transgenic tobacco (*Nicotiana tabacum*) overexpressing *CsMYB4a*, one of the MYB4/7/32 subgroup members in Tea plant (*Camellia sinensis*), displayed a series of auxin-deficient phenotypes (Ma et al., 2021, Preprint). Thus, our results provide a new insight into the relationship between lignin and auxin metabolism for the determination of rice leaf angle.

### Endogenous auxin may inhibit adaxial cell elongation at lamina joints *via* negatively regulating the transcription of *Expansin* and *XTH* genes

Deficiency in endogenous auxin stimulates the longitudinal elongation of adaxial cells at lamina joints, thereby accelerating leaf inclination (Zhao et al., 2013). However, the underlying mechanism is not well-understood. On the contrary, exogenous auxin treatment promotes expression of cell elongation-related genes, such as *Expansin* and *XTH* genes, which results in lamina joint bending (Yokoyama and Nishitani, 2001; Esmon et al., 2006; Nakamura et al., 2009); Expansins induce a pH-dependent relaxation of cell wall non-enzymatically, thus enabling cell elongation (Marowa et al., 2016), and the restructuring of wall-bound xyloglucan by XTHs allows cell expansion (Nishitani et al., 2006). Considering the opposite roles of endogenous and exogenous auxin in the determination of leaf angle (Nakamura et al., 2009; Zhao et al., 2013), we speculate that endogenous auxin at the lamina joints downregulates the transcription of these cell elongation-related genes. Indeed, we demonstrated that the expression of *Expansin* and *XTH* genes at the lamina joints is transcriptionally upregulated in *OsMYB7* overexpressors and downregulated in the *osmyb7-1* mutant (**Figure 7**). Moreover, OsMYB7 functions to accelerate cell elongation at the adaxial side of lamina joints (**Figure 8**), in agreement with the expression profiles. These data provide further information on the functional roles of endogenous auxin in controlling the adaxial cell elongation of lamina joints.

## Data availability statement

The raw data supporting the conclusions of this article will be made available by the authors, without undue reservation.

## Author contributions

N-CP conceived and supervised the project; S-HK performed the research with the help of JY, HK, and S-JL; S-HK, TK, and KK analyzed the data; S-HK and N-CP wrote the manuscript. All authors read and approved the final manuscript.
