# Supplementary material for: OsMYB7 determines leaf angle at the late developmental stage of lamina joints in rice
Source: Front Plant Sci. 2023 Apr 14;14:1167202. doi: 10.3389/fpls.2023.1167202 (PMC10140434; doi:10.3389/fpls.2023.1167202)
Supplement: Supplementary file 1 [file DataSheet_1.pdf]

Supplementary materials - Kim et al.

```
                                     R2 repeat of MYB DNA-binding domain
OsMYB7      1   MGRSPCCEKEHTNKGAWTKEEDERLVAYIRAHGEGCWRSLPKAAGLLRCGKSCRLRWINY
OsMYB108    1   MGRSPCCEKAHTNKGAWTKEEDDRLIAYIKAHGEGCWRSLPKAAGLLRCGKSCRLRWINY
ZmMYB42     1   MGRSPCCEKAHTNRGAWTKEEDERLVAYVRAHGEGCWRSLPRAAGLLRCGKSCRLRWINY
ZmMYB31     1   MGRSPCCEKAHTNKGAWTKEEDERLVAHIRAHGEGCWRSLPKAAGLLRCGKSCRLRWINY
AtMYB4      1   MGRSPCCEKAHTNKGAWTKEEDERLVAYIKAHGEGCWRSLPKAAGLLRCGKSCRLRWINY
AtMYB7      1   MGRSPCCEKEHMNKGAWTKEEDERLVSYIKSHGEGCWRSLPRAAGLLRCGKSCRLRWINY
AtMYB32     1   MGRSPCCEKDHTNKGAWTKEEDDKLISYIKAHGEGCWRSLPRSAGLQRCGKSCRLRWINY

                             R3 repeat of MYB DNA-binding domain
OsMYB7     61   LRPDLKRGNFTADEDDLIIKLHSLLGNKWSLIAARLPGRTDNEIKNYWNTHIRRKLLGRG
OsMYB108   61   LRPDLKRGNFTEEEDELIIKLHSLLGNKWSLIAGRLPGRTDNEIKNYWNTHIRRKLLSRG
ZmMYB42    61   LRPDLKRGNFTADEDDLIVKLHSLLGNKWSLIAARLPGRTDNEIKNYWNTHIRRKLLGSG
ZmMYB31    61   LRPDLKRGNFTEEEDELIVKLHSVLGNKWSLIAGRLPGRTDNEIKNYWNTHIRRKLLSRG
AtMYB4     61   LRPDLKRGNFTEEEDELIIKLHSLLGNKWSLIAGRLPGRTDNEIKNYWNTHIRRKLINRG
AtMYB7     61   LRPDLKRGNFTHDEDELIIKLHSLLGNKWSLIAARLPGRTDNEIKNYWNTHIKRKLLSKG
AtMYB32    61   LRPDLKRGNFTLEEDDLIIKLHSLLGNKWSLIATRLPGRTDNEIKNYWNTHVKRKLLRKG

OsMYB7    121   IDPVTHRPVNAA-AATISFHPQPPPTT---------------------------------
OsMYB108  121   IDPVTHRPIND-SASNITISFEAAAA--AARDDKAAVERREDH-PHQPKAVTVA------
ZmMYB42   121   IDPVTHRRVAGGAATTISFQPSPNSAAAAAAAETAAQA---------------------
ZmMYB31   121   IDPVTHRPVTEHHASNITISFETEVAAAARDDKKGAVERLEEE-EERNKATMVVGRDRQS
AtMYB4    121   IDPTSHRPIQESSASQDSKPTQLEPVTSNTIN---ISFTSAPKVETFHESISFPGKSE--
AtMYB7    121   IDPATHRGINEAKIS----DLKKTKDQIVKD----VSF------VTKFEETDKSGDQKQN
AtMYB32   121   IDPATHRPINETKTSQDSSDSSKTEDPLVKI----LSF------GPQLEKIANFGDERIQ

                                    EAR motif
OsMYB7    147   --------KEEQLILSKPPKCPDLNLDLCISPPSCQEEDD-----DYEAKPAMIVRAPE-
OsMYB108  171   ---QEQQAAADWGHG-KPLKCPDLNLDLCISLPSQEEPMMM-------------------
ZmMYB42   159   ------PIKAEETAAVKAPRCPDLNLDLCISPPCQHEDDGEEEDEELDLKPAFVKREALQ
ZmMYB31   180   QSQSHSHPAGEWGQGKRPLKCPDLNLDLCISPPCQEEEEMEEAA--------MRVR----
AtMYB4    176   -KISMLTFKEEKDECPVQEKKFPDLNLELRISLPDDVDRLQ-------------------
AtMYB7    167   KYIRNGLVCKEERVVVEEKIGPDLNLELRISPPWQNQR---------------------
AtMYB32   171   KRVE---------YSVVEERCLDLNLELRISPPWQDKLHDERN-----------------

OsMYB7    193   -LQRRRGGLCFGCSLGLQKECKCSGGGAGA-----------------GAGNNFLGLR--
OsMYB108  208   KPVKRETGVCFSCSLGLPKSTDCKC------------------------S--SFLGLR--
ZmMYB42   213   AGHGHGHGLCLGCGLGGQKGA----AGCSC-----------------SNGHHFLGLR--
ZmMYB31   228   PAVKREAGLCFGCSLGLPRTADCKC------------------------SSSSFLGLR--
AtMYB4    215   GHGKSTTPRCFKCSLGMINGMECRCGRMRCDVVGGSSKG------SDMSNGFDFLGLAKK
AtMYB7    205   -----EISTCTASRFYMENDMECSSETVKCQTENSSSISYSSIDISSSNVGYDFLGLK--
AtMYB32   205   LRFGRVKYRCSACRFGFGNGKECSCNNVKCQTEDSSSSSYSSTDISS-SIGYDFLGLN--

OsMYB7    232   --AGMLDFRSLPMK
OsMYB108  240   --TAMLDFRSLEMK
ZmMYB42   249   --TSVLDFRGLEMK
ZmMYB31   262   --TAMLDFRSLEMK
AtMYB4    269   ETTSLLGFRSLEMK
AtMYB7    258   --TRILDFRSLEMK
AtMYB32   262   -NTRVLDFSTLEMK
```

SUPPLEMENTARY FIGURE S1. Multiple sequence alignment of OsMYB7 homologs in *Oryza sativa*, *Zea mays*, and *Arabidopsis thaliana*. The amino acid sequences of OsMYB7 homologs acquired from the National Center for Biotechnology Information website (https://www.ncbi.nlm.nih.gov/) were subjected to protein sequence alignment using Clustal Omega from the EMBL-EBI website (https://www.ebi.ac.uk/Tools/msa/clustalo/) and the BoxShade 3.21 server (http://arete.ibb.waw.pl/PL/html/boxshade.html). Homologous regions are highlighted in black for identical amino acid residues or in gray for conservative amino acid substitutions. OsMYB108, ZmMYB42, ZmMYB31, AtMYB4, AtMYB7, and AtMYB32 show 65.9%, 66.2%, 60.8%, 55.7%, 54.7%, and 52.5% sequence similarity to OsMYB7, respectively, according to the NCBI-BLASTP program (https://blast.ncbi.nlm.nih.gov/Blast.cgi?PROGRAM=blastp&PAGE_TYPE=BlastSearch&LINK_LOC=blasthome). The conserved regions, such as R2 and R3 repeats of the MYB DNA-binding domain, and the EAR motif, are indicated by black lines. Numbers to the left side of each sequence represent amino acid positions. GenBank accession numbers of protein sequences are as follows: OsMYB7, XP_015650911; OsMYB108, XP_015612022; ZmMYB42, ADX60106; ZmMYB31, NP_001105949; AtMYB4, NP_195574; AtMYB7, NP_179263; AtMYB32, NP_195225. EAR, ethylene-responsive element binding factor-associated amphiphilic repression.

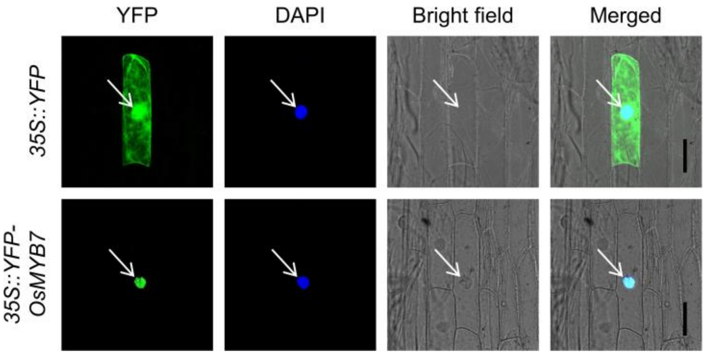

SUPPLEMENTARY FIGURE S2. Subcellular localization of OsMYB7. DAPI-stained onion epidermal cells expressing *YFP-OsMYB7* were observed using a confocal laser scanning microscope. Cells expressing *YFP* were used as a control. The white arrows point to the nucleus of each cell. Scale: 100 µm. Data shown are representatives of three independent experiments. DAPI, 4',6-diamidino-2-phenylindole; YFP, yellow fluorescent protein.

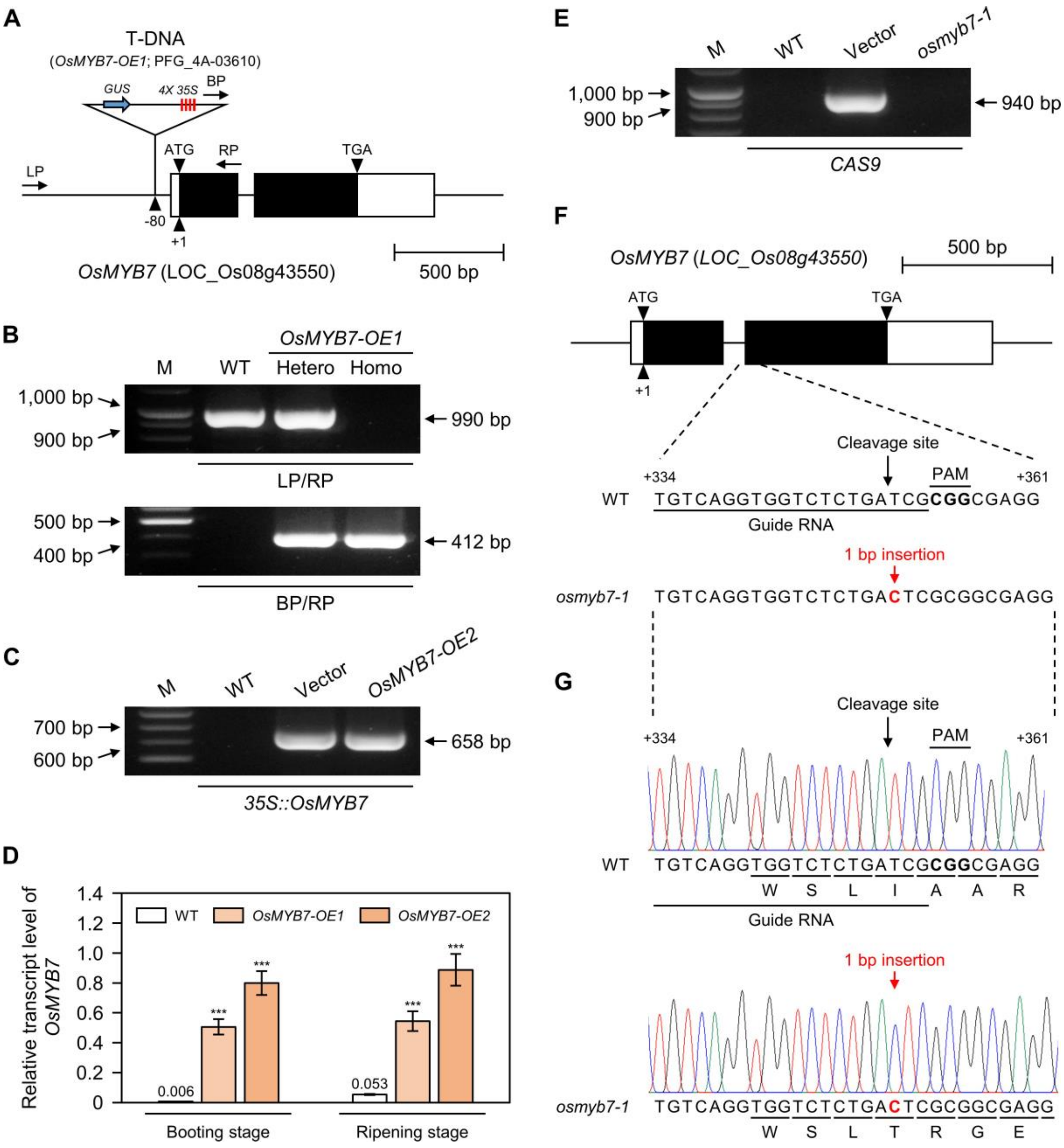

SUPPLEMENTARY FIGURE S3. Information of plant materials used in this study. **(A)** Schematic diagram illustrating the position of the T-DNA insertion in *OsMYB7-OE1*. Open and filled boxes indicate untranslated regions and exons, respectively. The position of the T-DNA insertion is shown relative to that of the translation initiation codon, which was set as +1. The promoterless *GUS* reporter gene and multimerized CaMV 35S enhancer harbored by the T-DNA are shown as the blue solid arrow and red vertical lines, respectively. Blac

k arrows indicate the primers used for PCR genotyping. BP, border primer; 4X 35S, tetra merized CaMV 35S enhancer; *GUS*, *β-glucuronidase*; LP, left primer; RP, right primer. **(B)** Identification of the T-DNA insertion in *OsMYB7-OE1*. Genomic DNA from the T_2 segregati ng population was isolated from individual plants and subjected to PCR using the primers denoted in **(A)**. LP + RP primers amplify the 990-bp WT allele; BP + RP primers amplify the 412-bp T-DNA fragment. Plants homozygous for the T-DNA insertion were selected an d used for further study. M, marker. **(C)** Confirmation of rice transformation in *OsMYB7-O E2* by genomic PCR. The *35S:OsMYB7* construct was introduced into rice callus to gener ate *OsMYB7-OE2*. Genomic DNA of the resulting T_0 plants was extracted, followed by PC R analysis using a primer set that amplifies part of the *35S:OsMYB7* construct. WT and t he *35S:OsMYB7* construct were used as a negative and positive control, respectively. M, marker. **(D)** Relative *OsMYB7* transcript levels in WT, *OsMYB7-OE1*, and *OsMYB7-OE2* pl ants. Total RNA isolated from flag leaf lamina joints of plants at the booting stage (105 D AS) or at the ripening stage (30 DAH) grown in a natural paddy field was subjected to R T-qPCR analysis, with *GAPDH* serving as a reference for normalization. Data are present ed as means ± SD (n = 4). Asterisks indicate significant differences compared to WT as determined by two-tailed Student's t-test (***P < 0.001). DAH, days after heading; DAS, d ays after sowing. **(E)** PCR verification of the T-DNA free *osmyb7-1* mutant. The T-DNA e ncoding both Cas9 protein and *OsMYB7*-targeted single guide RNA was introduced into ri ce callus to generate *osmyb7-1*. Genomic DNA was isolated for the resulting T_1 segregati ng population and subjected to PCR analysis using a primer set that amplifies part of *Ca s9* to obtain transgene-free *osmyb7-1* mutant plants for further study. WT and the vector containing *Cas9* were used as a negative and positive control, respectively. M, marker.
(F) Schematic diagram showing the position of the target site for *OsMYB7* gene editing d escribed in **(E)**. The 20-nt spacer region in *OsMYB7* is underlined, and the PAM is emph asized in bold. The location of the Cas9 cleavage site, 3-4 bp upstream of the PAM, is s hown as a black arrow. Nucleotide numbering is relative to the ATG start codon. PAM, pr otospacer adjacent motif. **(G)** Chromatograms of direct sequencing results from genomic P CR products of WT and the *osmyb7-1* mutant. The *OsMYB7* genomic DNA region around the target site illustrated in **(F)** was amplified, and the resulting PCR products were subje cted to direct Sanger sequencing.

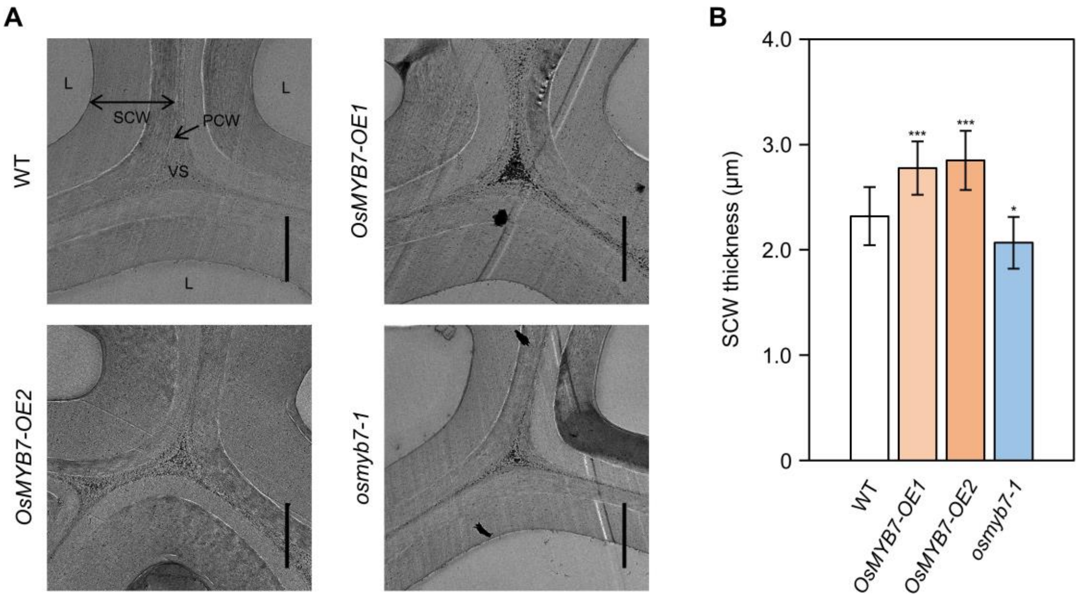

SUPPLEMENTARY FIGURE S4. Transmission electron microscopic analysis of the lamina joint. **(A)** Secondary cell walls of sclerenchyma cells at lamina joints. Collars of flag leaves from WT, *OsMYB7-OE1*, *OsMYB7-OE2*, and *osmyb7-1* plants at the ripening stage (30 DAH) grown under natural day-night conditions were sampled for examination. Scale: 2 μm. Images shown are representatives of four independent experiments. L, lumen; PCW, primary cell wall; SCW, secondary cell wall; VS, void space. **(B)** Quantitative data of the secondary cell wall thickness in **(A)**. Data are presented as means ± SD from at least ten cells. Asterisks denote significant differences, as determined by two-tailed Student's *t*-test (*P < 0.05 and ***P < 0.001).

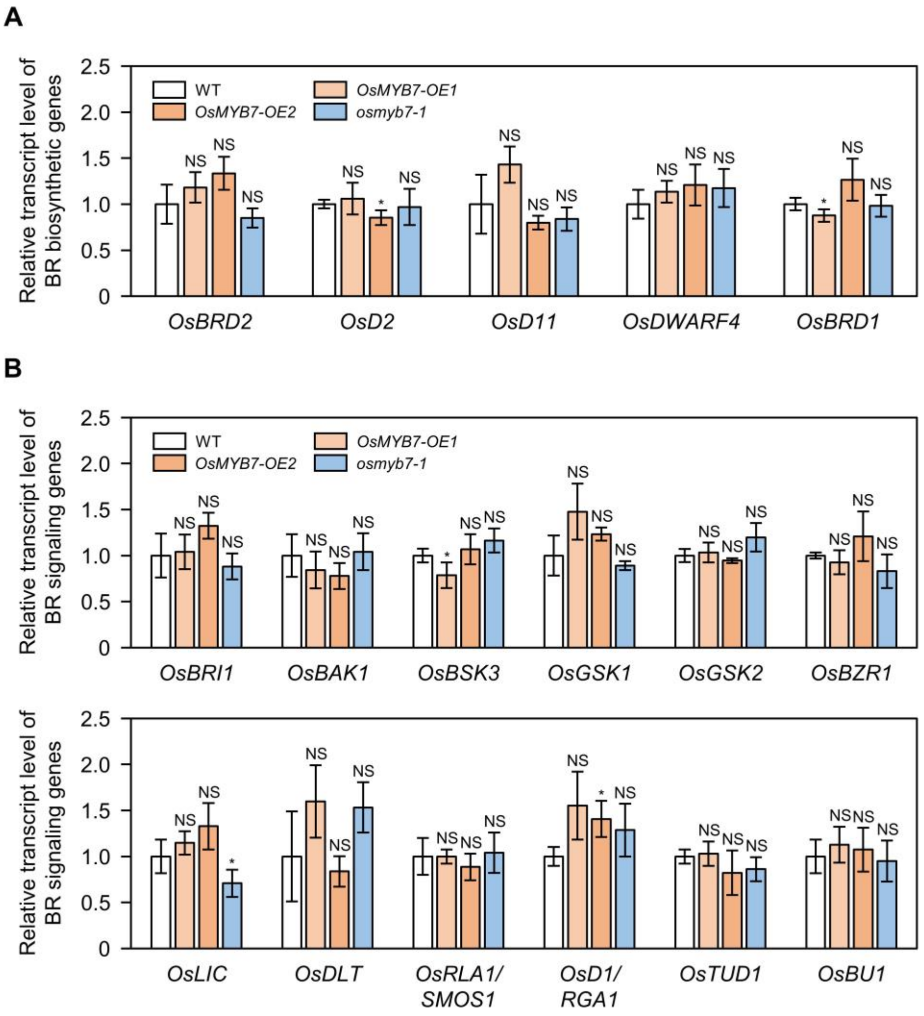

SUPPLEMENTARY FIGURE S5. Transcript levels of representative BR-related genes that regulate leaf inclination. **(A, B)** Relative expression levels of BR biosynthetic **(A)** and signaling **(B)** genes in WT, *OsMYB7-OE1*, *OsMYB7-OE2*, and *osmyb7-1* plants. Samples harvested in **Figure 4A** were subjected to RT-qPCR analysis, using *GAPDH* as an internal control. The normalized transcript levels of each gene are presented relative to those in WT, which were set to 1. Data are presented as means ± SD of four biological replicates. Asterisks indicate significant differences compared to WT as determined by two-tailed Student's *t*-test; $^*P < 0.05$. These experiments were repeated twice with similar results. NS, not significant.

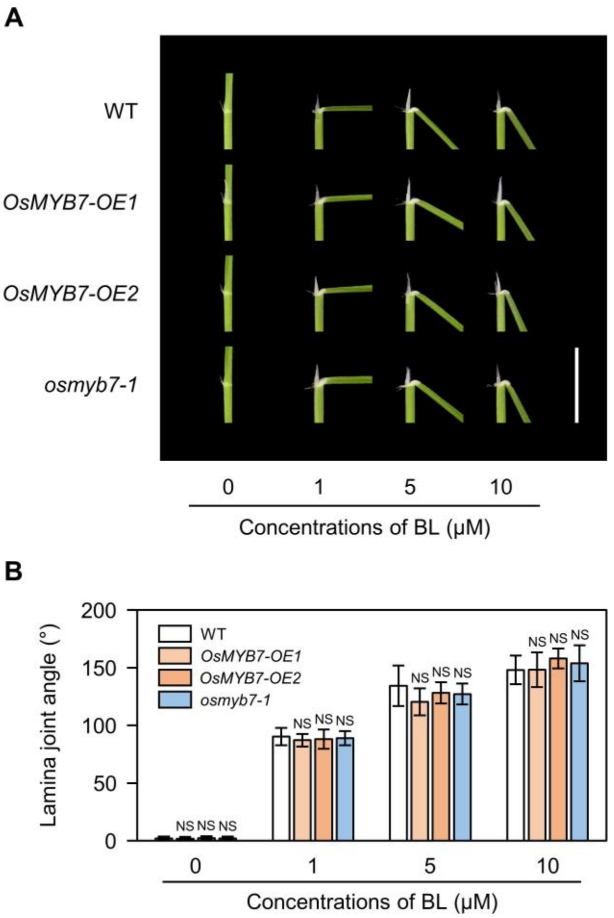

SUPPLEMENTARY FIGURE S6. BR-induced lamina joint inclination assay. **(A)** Effects of BL on the lamina inclination of WT, *OsMYB7-OE1*, *OsMYB7-OE2*, and *osmyb7-1*. Plants were grown in paddy soil for 10 days under long-day conditions (14.5 h light, 30°C / 9.5 h dark, 24°C) with 60% relative humidity in an artificial growth chamber. Approximately 2-cm of lamina joint segments, consisting of leaf blade, lamina joint at S4 developmental stage, and leaf sheath, were excised from uniform seedlings, followed by incubation in distilled water containing 0, 1, 5, or 10 μM of BL under dark conditions at 30°C for 48 h. The excised segments were kept in a vertical orientation to minimize gravitropic response of the lamina joint. Photographs of representative lamina joint segments for each group were taken. Scale: 1 cm. **(B)** Lamina joint angle shown in **(A)**. Degrees of the leaf blade angle against the axis of leaf sheath were measured with a protractor. Data are presented as means ± SD from ten lamina joint segments. Statistical analysis using two-tailed Student's *t*-test revealed no obvious differences in lamina inclination of *OsMYB7-OE1*, *OsMYB7-*

OE2, and *osmyb7-1* segments compared to that of WT segments. These experiments were performed twice yielding similar results. BL, 24-epibrassinolide; NS, not significant.

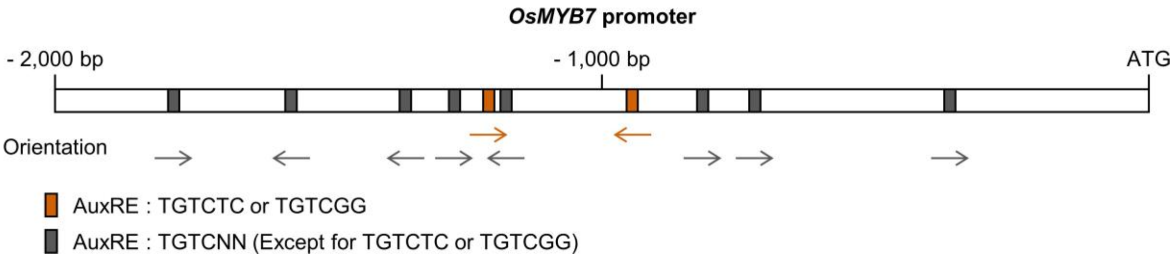

SUPPLEMENTARY FIGURE S7. Identification of putative AuxREs in the promoter of *OsMYB7*. Sequence of the *OsMYB7* promoter (−2,000 bp to −1 bp from the initiation codon) obtained from the Gramene website (https://www.gramene.org/) is shown as a schematic diagram. Positions and orientations of the AuxREs, TGTCNN consensus core sequences, are presented as filled boxes and arrows, respectively, in gray. The canonical AuxREs, such as TGTCTC and TGTCGG, are highlighted in yellow red. AuxRE, auxin-responsive element.

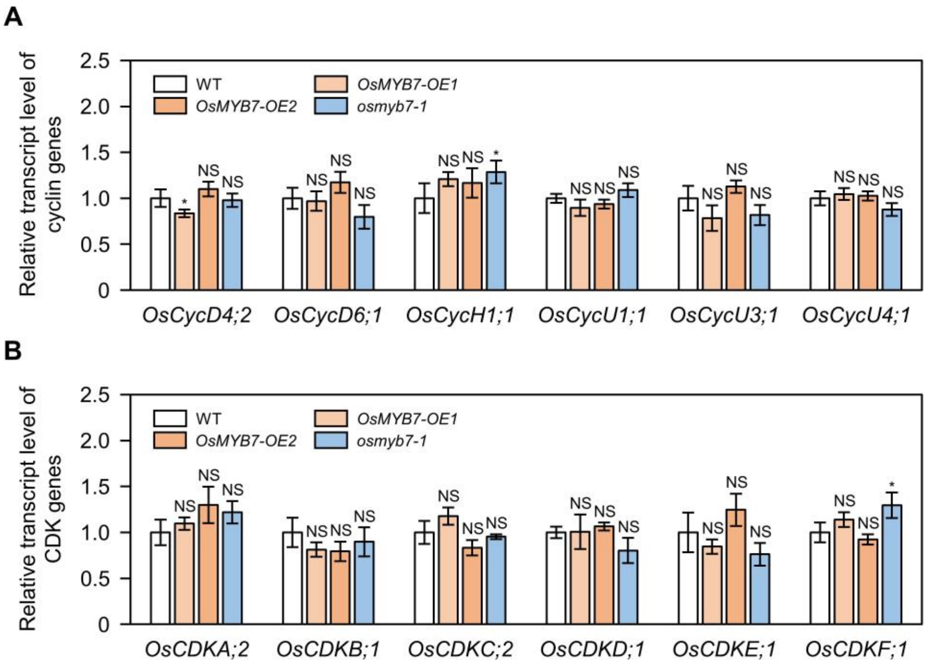

SUPPLEMENTARY FIGURE S8. Expression profiles of cell division-related genes at lamina joints of WT, *OsMYB7-OE1*, *OsMYB7-OE2*, and *osmyb7-1* plants. **(A, B)** Relative transcript levels of cyclin **(A)** and CDK **(B)** genes. The cDNA samples in **Figure 4A** were subjected to RT-qPCR analysis, with *GAPDH* used as reference for normalization, and shown relative to those in WT, which were set to 1. Data are presented as means ± SD from four independent biological replicates. Asterisks indicate significant differences as determined by two-tailed Student's *t*-test (**P* < 0.05). These experiments were performed twice yielding similar results. CDK, cyclin-dependent kinase; NS, not significant.

SUPPLEMENTARY TABLE S1. *In silico* analysis for subcellular localization of OsMYB7 homologs.

	Plastid	Cytoplasm	Extra-cellular	Nucleus	Mito-chondrion	Cell Membrane	Endo-plasmic Reticulum
**OsMYB7**	0.0745 (7.4%)	0.229 (23%)	0.00249 (0.25%)	0.431 (43%)	0.0736 (7.4%)	0.0523 (5.2%)	0.015 (1.5%)
**OsMYB108**	0.00797 (0.80%)	0.0159 (1.6%)	0.00032 (0.032%)	0.864 (86%)	0.00485 (0.48%)	0.0414 (4.1%)	0.0092 (0.92%)
**ZmMYB42**	0.0349 (3.5%)	0.0256 (2.6%)	0.00122 (0.12%)	0.684 (68%)	0.0128 (1.3%)	0.147 (15%)	0.0105 (1.0%)
**ZmMYB31**	0.00562 (0.56%)	0.0232 (2.3%)	0.000544 (0.054%)	0.799 (80%)	0.00551 (0.55%)	0.0367 (3.7%)	0.0166 (1.7%)
**AtMYB4**	0.0424 (4.2%)	0.0847 (8.5%)	0.000841 (0.084%)	0.729 (73%)	0.016 (1.6%)	0.0324 (3.2%)	0.00888 (0.89%)
**AtMYB7**	0.0279 (2.8%)	0.0457 (4.6%)	0.000357 (0.036%)	0.716 (72%)	0.00311 (0.31%)	0.0365 (3.6%)	0.0217 (2.2%)
**AtMYB32**	0.0206 (2.1%)	0.0316 (3.2%)	0.000219 (0.022%)	0.822 (82%)	0.00181 (0.18%)	0.0139 (1.4%)	0.0141 (1.4%)

	Golgi Apparatus	Vacuole	Peroxisome	Cell Wall	Mito-chondrion / Plastid	Cytoplasm / Nucleus	Cytoplasm / Golgi Apparatus
**OsMYB7**	0.0194 (1.9%)	0.00741 (0.74%)	0.0229 (2.3%)	0.00237 (0.24%)	0.00586 (0.59%)	0.0606 (6.1%)	0.00348 (0.35%)
**OsMYB108**	0.00867 (0.87%)	0.00102 (0.10%)	0.0103 (1.0%)	0.00037 (0.037%)	0.00137 (0.14%)	0.0332 (3.3%)	0.00107 (0.11%)
**ZmMYB42**	0.0243 (2.4%)	0.00477 (0.48%)	0.013 (1.3%)	0.000641 (0.064%)	0.00563 (0.56%)	0.0336 (3.4%)	0.00197 (0.20%)
**ZmMYB31**	0.00848 (0.85%)	0.00156 (0.16%)	0.00918 (0.92%)	0.000563 (0.056%)	0.00225 (0.22%)	0.0898 (9.0%)	0.000912 (0.091%)
**AtMYB4**	0.00912 (0.91%)	0.00198 (0.20%)	0.0101 (1.0%)	0.000784 (0.078%)	0.00229 (0.23%)	0.0605 (6.0%)	0.00115 (0.12%)
**AtMYB7**	0.00352 (0.35%)	0.0026 (0.26%)	0.00395 (0.40%)	0.000663 (0.066%)	0.000577 (0.058%)	0.137 (14%)	0.000889 (0.089%)
**AtMYB32**	0.0086 (0.86%)	0.000875 (0.088%)	0.00366 (0.37%)	0.000235 (0.024%)	0.000246 (0.025%)	0.0812 (8.1%)	0.000655 (0.066%)

The protein sequences of OsMYB7 homologs in **Supplementary Figure S1** were analyzed using the PseAAC-NCC-DIPEP prediction module of Plant-mSubP program (http://bioinfo.usu.edu/Plant-mSubP/), one of the *in silico* analysis tools for subcellular localization of proteins in plant cell. Values in the table represent likelihoods of localization to the corresponding organelles. All the OsMYB7 homologs were predicted to be nuclear proteins with the highest localization likelihoods, and the values of nucleus for each protein were highlighted in red letters.

SUPPLEMENTARY TABLE S2. Six developmental stages of the lamina joint.

Stage	Morphological features	Representative image	Reference
S1 (Initiation)	> Lamina joint differentiation is initiated<br>> Lamina joint is transparent and hollow	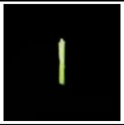	Zhou, L. J., Xiao, L. T., Xue, H. W. (2017). Dynamic cytology and transcriptional regulation of rice lamina joint development. *Plant Physiol*. 174, 1728-1746. doi: 10.1104/pp. 17.00413
S2 (Young)	> Lamina joint protrudes and becomes larger<br>> Lamina joint is white or creamy yellow	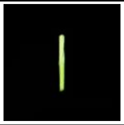	
S3 (Young)	> Lamina joint is still enclosed by leaf sheath<br>> A ligule and a pair of auricles can be observed	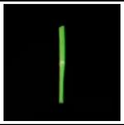	
S4 (Maturation)	> Lamina joint emerges from leaf sheath<br>> Leaf blade and leaf sheath fully develope	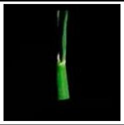	
S5 (Post-maturation)	> Asymmetric cell elongation and/or division between the abaxial and adaxial sides lead to increased leaf angle	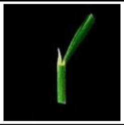	
S6 (Senescence)	> Lamina joint reaches the maximum angle<br>> Lamina joint begins to wither due to water loss	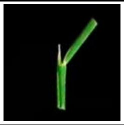	

SUPPLEMENTARY TABLE S3. Genes described in this study and their corresponding locus IDs.

A. *OsMYB7* and *GAPDH*

Gene	MSU_Locus	RAP_Locus	Accession No.	Reference
OsMYB7	LOC_Os08g43550	Os08g0549000	CI473176	Miyamoto et al., 2019
GAPDH	LOC_Os04g40950	Os04g0486600	AK064960	Jain et al., 2006

B. *OsbHLH079* and *ONAC026*

Gene	MSU_Locus	RAP_Locus	Accession No.	Reference
OsbHLH079	LOC_Os02g47660	Os02g0705500	AK119183	Seo et al., 2020
ONAC026	LOC_Os01g29840	Os01g0393100	AK107407	Mathew et al., 2016

C. Lignin biosynthetic genes

Gene	MSU_Locus	RAP_Locus	Accession No.	Reference
OsPAL1	LOC_Os02g41630	Os02g0626100	AK060724	Tonnessen et al., 2015
OsPAL2	LOC_Os02g41650	Os02g0626400	AK060724	
OsC4H2	LOC_Os05g25640	Os05g0320700	AK104994	Yang et al., 2005
Os4CL3	LOC_Os02g08100	Os02g0177600	AK070083	Gui et al., 2011
OsHCT1	LOC_Os04g42250	Os04g0500700	AK072528	Kim et al., 2012
OsHCT2	LOC_Os02g39850	Os02g0611800	AK104319	
OsC3H	LOC_Os05g41440	Os05g0494000	AK099695	Takeda et al., 2018
OsCOA1	LOC_Os06g06980	Os06g0165800	AK065744	Zhao et al., 2004
OsCOA20	LOC_Os08g38900	Os08g0498100	AK104326	
OsCCoAOMT1	LOC_Os08g38910	Os08g0498400	AK061757	Lee et al., 2008
OsCOMT1	LOC_Os08g06100	Os08g0157500	AK064768	Hamberger et al., 2008
OsCCR17	LOC_Os09g04050	Os09g0127300	AK100234	Park et al., 2017
OsCCR19	LOC_Os09g25150	Os09g0419200	AK104860	
OsCCR20	LOC_Os08g34280	Os08g0441500	AK072872	
OsCAD2	LOC_Os02g09490	Os02g0187800	AK105011	Park et al., 2018
OsCAD6	LOC_Os04g15920	Os04g0229100	AK099270	

D. Cellulose biosynthetic genes

Gene	MSU_Locus	RAP_Locus	Accession No.	Reference
OsCESA1	LOC_Os05g08370	Os05g0176100	AK099281	Wang et al., 2010
OsCESA3	LOC_Os07g24190	Os07g0424400	AK120236	
OsCESA5	LOC_Os03g62090	Os03g0837100	AK100877	
OsCESA6	LOC_Os07g14850	Os07g0252400	AK100914	
OsCESA8	LOC_Os07g10770	Os07g0208500	AK072356	
OsCSLA1	LOC_Os02g09930	Os02g0192500	AK059580	
OsCSLA6	LOC_Os02g51060	Os02g0744600	AK058756	
OsCSLC1	LOC_Os01g56130	Os01g0766900	AK110759	
OsCSLC7	LOC_Os05g43530	Os05g0510800	AF435642	
OsCSLC9	LOC_Os03g56060	Os03g0770800	AK121805	
OsCSLD2	LOC_Os06g02180	Os06g0111800	AK105393	
OsCSLE1	LOC_Os09g30120	Os09g0478100	AK102766	
OsCSLF6	LOC_Os08g06380	Os08g0160500	AK109812	
OsCSLH1	LOC_Os10g20090	Os10g0341700	AK121003	

E. Brassinosteroid (BR) biosynthetic genes

Gene	MSU_Locus	RAP_Locus	Accession No.	Reference
OsBRD2	LOC_Os10g25780	Os10g0397400	AK111949	Castorina and Consonni, 2020
OsD2	LOC_Os01g10040	Os01g0197100	C97895	
OsD11	LOC_Os04g39430	Os04g0469800	AK106528	
OsDWARF4	LOC_Os03g12660	Os03g0227700	CI552150	
OsBRD1	LOC_Os03g40540	Os03g0602300	AK072295	

F. Brassinosteroid (BR) signaling genes

Gene	MSU_Locus	RAP_Locus	Accession No.	Reference
OsBRI1	LOC_Os01g52050	Os01g0718300	AK101085	Xu et al., 2021
OsBAK1	LOC_Os08g07760	Os08g0174700	AK103038	
OsBSK3	LOC_Os04g58750	Os04g0684200	AK101506	
OsGSK1	LOC_Os01g10840	Os01g0205700	AK099863	
OsGSK2	LOC_Os05g11730	Os05g0207500	AK102147	
OsBZR1	LOC_Os07g39220	Os07g0580500	AK106748	
OsLIC	LOC_Os06g49080	Os06g0704300	AK107008	

OsDLT	LOC_Os06g03710	Os06g0127800	AK106449	
OsRLA1/SMOS1	LOC_Os05g32270	Os05g0389000	AK059324	
OsD1/RGA1	LOC_Os05g26890	Os05g0333200	D38232	
OsTUD1	LOC_Os03g13010	Os03g0232600	AK068218	
OsBU1	LOC_Os06g12210	Os06g0226500	AK071601	
**G. Auxin biosynthetic genes**				
**Gene**	**MSU_Locus**	**RAP_Locus**	**Accession No.**	**Reference**
OsTAA1	LOC_Os01g07500	Os01g0169800	AK061054	Yoshikawa et al., 2014
OsYUCCA3	LOC_Os01g53200	Os01g0732700	AP014957	
OsYUCCA4	LOC_Os01g12490	Os01g0224700	AK070386	
OsYUCCA5	LOC_Os12g32750	Os12g0512000	C98496	Zhang et al., 2018
OsYUCCA6	LOC_Os07g25540	Os07g0437000	CI249850	
OsYUCCA7	LOC_Os04g03980	Os04g0128900	AK068976	
**H. Auxin conjugation genes**				
**Gene**	**MSU_Locus**	**RAP_Locus**	**Accession No.**	**Reference**
OsGH3-1	LOC_Os01g57610	Os01g0785400	AK063368	
OsGH3-3	LOC_Os01g12160	Os01g0221100	AK072125	
OsGH3-4	LOC_Os05g42150	Os05g0500900	AK101932	Terol et al., 2006
OsGH3-6	LOC_Os05g05180	Os05g0143800	AK106538	
OsGH3-7	LOC_Os06g30440	Os06g0499500	AK107353	
OsDAO	LOC_Os04g39980	Os04g0475600	AK105400	Zhao et al., 2013
**I. Cyclin genes**				
**Gene**	**MSU_Locus**	**RAP_Locus**	**Accession No.**	**Reference**
OsCycD4;2	LOC_Os08g37390	Os08g0479300	AK070025	
OsCycD6;1	LOC_Os07g37010	Os07g0556000	AK121938	
OsCycH1;1	LOC_Os03g52750	Os03g0737600	AK101854	
OsCycU1;1	LOC_Os04g53680	Os04g0628900	AP014960	La et al., 2006
OsCycU3;1	LOC_Os05g33040	Os05g0398000	AK070478	
OsCycU4;1	LOC_Os10g41430	Os10g0563900	AK107529	
**J. Cyclin-dependent kinase (CDK) genes**				
**Gene**	**MSU_Locus**	**RAP_Locus**	**Accession No.**	**Reference**
OsCDKA;2	LOC_Os02g03060	Os02g0123100	AK101344	
OsCDKB;1	LOC_Os01g67160	Os01g0897000	CI522617	
OsCDKC;2	LOC_Os01g72790	Os01g0958000	AK103469	Guo et al., 2007
OsCDKD;1	LOC_Os05g32600	Os05g0392300	AK120162	
OsCDKE;1	LOC_Os10g42950	Os10g0580300	AK066824	
OsCDKF;1	LOC_Os06g22820	Os06g0334400	AK059487	
**K. Expansin genes**				
**Gene**	**MSU_Locus**	**RAP_Locus**	**Accession No.**	**Reference**
OsEXPA1	LOC_Os04g15840	Os04g0228400	AK069548	
OsEXPA4	LOC_Os05g39990	Os05g0477600	AK100179	
OsEXPA6	LOC_Os03g21820	Os03g0336400	AK107698	
OsEXPA10	LOC_Os04g49410	Os04g0583500	AK066414	
OsEXPB3	LOC_Os10g40720	Os10g0555900	AK100959	Sampedro et al., 2005
OsEXPB4	LOC_Os10g40730	Os10g0556100	AK060096	
OsEXPB6	LOC_Os10g40700	Os10g0555600	AK105799	
OsEXLA2	LOC_Os10g39640	Os10g0542400	AK068088	
OsEXLA3	LOC_Os07g29290	Os07g0475400	AK102489	
**L. *Xyloglucan endotransglucosylase/hydrolase* (*XTH*) genes**				
**Gene**	**MSU_Locus**	**RAP_Locus**	**Accession No.**	**Reference**
OsXTH9	LOC_Os04g51460	Os04g0604300	AF443603	
OsXTH10	LOC_Os06g48200	Os06g0697000	AK105513	
OsXTH11	LOC_Os06g48160	Os06g0696400	AK058291	
OsXTH12	LOC_Os06g48180	Os06g0696600	AK105934	
OsXTH15	LOC_Os06g22919	Os06g0335900	AK120283	Yokoyama et al., 2004
OsXTH17	LOC_Os08g13920	Os08g0237000	AK060654	
OsXTH21	LOC_Os07g29750	Os07g0480800	CI419522	
OsXTH23	LOC_Os02g46910	Os02g0696500	AK111242	
OsXTH28	LOC_Os03g13570	Os03g0239000	AK061284	
**M. References**				

Castorina, G., and Consonni, G. (2020). The role of brassinosteroids in controlling plant height in *Poaceae*: a genetic perspective. *Int. J. Mol. Sci*. 157, 574-586. doi: 10.3390/ijms21041191

Gui, J., Shen, J., Li, L. (2011). Functional characterization of evolutionarily divergent 4-coumarate:coenzyme A ligases in rice. *Plant Physiol*. 157, 574-586. doi: 10.1104/pp.111.178301

Guo, J., Song, J., Wang, F., Zhang, X. S. (2007). Genome-wide identification and expression analysis of rice cell cycle genes. *Plant Mol. Biol*. 64, 349-360. doi: 10.1007/s11103-007-9154-y

Hamberger, B., Ellis, M., Friedmann, M., de Azevedo Souza, C., Barbazuk, B., Douglas, C. J. (2008). Genome-wide analyses of phenylpropanoid-related genes in *Populus trichocarpa*, *Arabidopsis thaliana*, and *Oryza sativa*: the *Populus* lignin toolbox and conservation and diversification of angiosperm gene families. *Botany* 85, 1182-1201. doi: 10.1139/B07-098

Jain, M., Nijhawan, A., Tyagi, A. K., Khurana, J. P. (2006). Validation of housekeeping genes as internal control for studying gene expression in rice by quantitative real-time PCR. *Biochem. Biophys. Res. Commun*. 345, 646-651. doi: 10.1016/j.bbrc.2006.04.140

Kim, I. A., Kim, B. G., Kim, M., Ahn, J. H. (2012). Characterization of hydroxycinnamoyltransferase from rice and its application for biological synthesis of hydroxycinnamoyl glycerols. *Phytochemistry* 76, 25-31. doi: 10.1016/j.phytochem.2011.12.015

La, H., Li, J., Ji, Z., Cheng, Y., Li, X., Jiang, S., et al. (2006). Genome-wide analysis of cyclin family in rice (*Oryza Sativa* L.). *Mol. Genet. Genom*. 275, 374-386. doi: 10.1007/s00438-005-0093-5

Lee, Y. J., Kim, B. G., Chong, Y., Lim, Y., Ahn, J. H. (2008). Cation dependent *O*-methyltransferases from rice. *Planta* 227, 641-647. doi: 10.1007/s00425-007-0646-4

Mathew, I. E., Das, S., Mahto, A., Agarwal, P. (2016). Three rice NAC transcription factors heteromerize and are associated with seed size. *Front Plant Sci*. 7, 1638. doi: 10.3389/fpls.2016.01638

Miyamoto, T., Takada, R., Tobimatsu, Y., Takeda, Y., Suzuki, S., Yamamura, M., et al. (2019). OsMYB108 loss-of-function enriches *p*-coumaroylated and tricin lignin units in rice cell walls. *Plant J*. 98, 975-987. doi: 10.1111/tpj.14290

Park, H. L., Bhoo, S. H., Kwon, M., Lee, S. W., Cho, M. H. (2017). Biochemical and expression analyses of the rice cinnamoyl-CoA reductase gene family. *Front. Plant Sci*. 8, 2099. doi: 10.3389/fpls.2017.02099

Park, H. L., Kim, T. L., Bhoo, S. H., Lee, T. H., Lee, S. W., Cho, M. H. (2018). Biochemical characterization of the rice cinnamyl alcohol dehydrogenase gene family. *Molecules* 23, 2659. doi: 10.3390/molecules23102659

Sampedro, J., Lee, Y., Carey, R. E., DePamphilis, C., Cosgrove, D. J. (2005). Use of genomic history to improve phylogeny and understanding of births and deaths in a gene family. *Plant J*. 44, 409-419. doi: 10.1111/j.1365-313X.2005.02540.x

Seo, H., Kim, S. H., Lee, B. D., Lim, J. H., Lee, S. J., An, G., et al. (2020). The rice *basic Helix-Loop-Helix 79* (*OsbHLH079*) determines leaf angle and grain shape. *Int. J. Mol. Sci.* 21, 2090. doi: 10.3390/ijms21062090

Takeda, Y., Tobimatsu, Y., Karlen, S. D., Koshiba, T., Suzuki, S., Yamamura, M., et al. (2018). Downregulation of p-*COUMAROYL ESTER 3-HYDROXYLASE* in rice leads to altered cell wall structures and improves biomass saccharification. *Plant J*. 95, 796-811. doi: 10.1111/tpj.13988

Terol, J., Domingo, C., Talón, M. (2006). The GH3 family in plants: genome wide analysis in rice and evolutionary history based on EST analysis. *Gene* 371, 279-290. doi: 10.1016/j.gene.2005.12.014

Tonnessen, B. W., Manosalva, P., Lang, J. M., Baraoidan, M., Bordeos, A., Mauleon, R., et al. (2015). Rice phenylalanine ammonia-lyase gene *OsPAL4* is associated with broad spectrum disease resistance. *Plant Mol. Biol*. 87, 273-286. doi: 10.1007/s11103-014-0275-9

Wang, L., Guo, K., Li, Y., Tu, Y., Hu, H., Wang, B., et al. (2010). Expression profiling and integrative analysis of the *CESA*/*CSL* superfamily in rice. *BMC Plant Biol*. 10, 282. doi: 10.1186/1471-2229-10-282

Xu, J., Wang, J. J., Xue, H. W., Zhang, G. H. (2021). Leaf direction: lamina joint development and environmental responses. *Plant Cell Environ*. 44, 2441-2454. doi: 10.1111/pce.14065

Yang, D. H., Chung, B. Y., Kim, J. S., Kim, J. H., Yun, P. Y., Lee, Y. K., et al. (2005). cDNA cloning and sequence analysis of the rice Cinnamate-4-Hydroxylase gene, a cytochrome P450-dependent monooxygenase involved in the general phenylpropanoid pathway. *J. Plant Biol*. 48, 311-318. doi: 10.1007/BF03030528

Yokoyama, R., Rose, J. K. C., Nishitani, K. (2004). A surprising diversity and abundance of xyloglucan endotransglucosylase/hydrolases in rice. Classification and expression analysis. *Plant Physiol*. 134, 1088-1099. doi: 10.1104/pp.103.035261

Yoshikawa, T., Ito, M., Sumikura, T., Nakayama, A., Nishimura, T., Kitano, H., et al. (2014). The rice *FISH BONE* gene encodes a tryptophan aminotransferase, which affects pleiotropic auxin-related processes. *Plant J*. 78, 927-936. doi: 10.1111/tpj.12517

Zhang, T., Li, R., Xing, J., Yan, L., Wang, R., Zhao, Y. (2018). The YUCCA-Auxin-WOX11 module controls crown root development in rice. *Front. Plant Sci*. 9, 523. doi: 10.3389/fpls.2018.00523

Zhao, H., Sheng, Q., Lü, S., Wang, T., Song, Y. (2004). Characterization of three rice *CCoAOMT* genes. *Chin. Sci. Bull*. 49, 1602-1606. doi: 10.1007/BF03184129

Zhao, Z., Zhang, Y., Liu, X., Zhang, X., Liu, S., Yu, X., et al. (2013). A role for a dioxygenase in auxin metabolism and reproductive development in rice. *Dev. Cell* 27, 113-122. doi: 10.1016/j.devcel.2013.09.005

SUPPLEMENTARY TABLE S4. Primers used in this study.

**A. Cloning**		
**Primer name**	**Forward primer (5' to 3')**	**Reverse primer (5' to 3')**
OsMYB7 coding sequence	ATGGGGAGGTCGCCGTGCTGCGA	TCATTTCATGGGGAGGCTTCTGA
osmyb7-1 guide RNA	GGCATGTCAGGTGGTCTCTGATCG	AAACCGATCAGAGACCACCTGACA
**B. Genotyping**		
**Primer name**	**Forward primer (5' to 3')**	**Reverse primer (5' to 3')**
OsMYB7-OE1 LP/RP	TCTAAACATTCGAGGTGACCG	GTTACTTGTTGCCGAGGAGG
OsMYB7-OE1 BP/RP	CGTCCGCAATGTGTTATTAAG	GTTACTTGTTGCCGAGGAGG
35S::OsMYB7	CTATCCTTCGCAAGACCCTT	ATGCAGAGGTCCAGGTTGA
CAS9	CTGTAGAGTCCTGTTGTCAAAAT	AACTGAAGGCGGGAAACGACAAT
**C. Transactivation / Transrepression activity assay**		
**Primer name**	**Forward primer (5' to 3')**	**Reverse primer (5' to 3')**
OsMYB7-pGBKT7	GTCGACTCATGGGGAGGTCGCCGTGC	GCGGCCGCTCATTTCATGGGGAGGCT
OsbHLH079-pGBKT7	GAACGGGTGAGGAGGGAGAGGATCAG	GCGGCCGCTTACATTTCCATTTTGAGA
rGAL4	CCATGGGAGCCAATTTTAATCAAAGTG	GAATTCCTCTTTTTTTGGGTTTGGTGG
OsMYB7-rGAL4	GTCGACTCATGGGGAGGTCGCCGTGC	GCGGCCGCTCATTTCATGGGGAGGCT
ONAC026-rGAL4	GTCGACTCATGGGAGAGCAGCAGCAG	GCGGCCGCTCAGTACTTCCAGATGGT
**D. RT-qPCR**		
**Primer name**	**Forward primer (5' to 3')**	**Reverse primer (5' to 3')**
GAPDH qPCR	AAGCCAGCATCCTATGATCAGATT	CGTAACCCAGAATACCCTTGAGTTT
OsMYB7 qPCR	CTCGGCAACAAGTGGTCTCTGAT	GATCCCCCTGCCGAGAAGCTT
OsPAL1 qPCR	CCGCTTCGTGTATCTTCAGAC	CAGCTAACACAAAGAACACGAGA
OsPAL2 qPCR	GACGTATAGCAACACAAAACTG	GAAACAGCAACAGTAACATCAAG
OsC4H2 qPCR	GGTAGTTATGTGTGTGTTCTG	TGAACCAACAAGTATAAGAAAAAAA
Os4CL3 qPCR	GAGATATGATGTTGCTGTCCA	TTTTATGAACATTGCACAAGCTG
OsHCT1 qPCR	CAGGCTGAGCACATGGAGAA	CTCTAGCTCTACAACTTCTCCCT
OsHCT2 qPCR	GATTCCACACGTTAGTTCCTGC	CAAAATGTGTCCTGCCCAAAAGC
OsC3H qPCR	TGCCTTGTGAATGAACGAATC	CATCGCTTGTCGTGTTAATCA
OsCOA1 qPCR	CGATGCCCAAGAACTAGTCA	ATAACATTTTCCAGTAGCTTGCAA
OsCOA20 qPCR	CTCTTCTACTGCTACAACTATAC	GTAGTACAGTAACAACCATCATC
OsCCoAOMT1 qPCR	TAGCCCCAAGACCCTCCTCAA	ATAGGTGTGCTCGCTGGTGAT
OsCOMT1 qPCR	CGTGGGTAATCATGTCGTTTG	TTAGAACTCAGAATCACCGGAAT
OsCCR17 qPCR	CTGCTGGCTGCTGATATATACTC	TGTCGATCGGTGTGCATGTAG
OsCCR19 qPCR	AGTGTTAGGCATCTGTTGTTA	CACGTCTGTTTATATTCAATGAT
OsCCR20 qPCR	GAGCATGAGGAAAACAGCAGA	CTACTTTGGTTTTACAGCACGG
OsCAD2 qPCR	CTTGAACTTGTTTGTGTGAGACTC	TGGTCCATATATTGCGAGGC
OsCAD6 qPCR	TTTAATTTATTGGAGGCTCTGCA	TTCTCGAATAAGTACAAAGTGCG
OsCESA1 qPCR	TCATGGGCAGGCAGAACCGC	CAGTTCACACCGCATTGCCCCA
OsCESA3 qPCR	ATCGGTGTGTGCTGAAGGAATAC	GAAGTTCACAAGGTTGCCGCA
OsCESA5 qPCR	GGAATGGATCTGCCGTCTGGA	AGGAACAAGGAATGAACAAGCCC
OsCESA6 qPCR	CAGCCTACACTCCATATATGCGG	TGGAACAAAAGAAATGCCGGAGAT
OsCESA8 qPCR	TGCCAGTTGTGTTTTTTTCAGAATAC	TATTTTCTGGTCTGTACGTAGCTGT
OsCSLA1 qPCR	GCCTTTTTCTCTGTTATGTGCTATTGT	CTCCTTGCCCAGATCACACC
OsCSLA6 qPCR	ATGCTATGACTACTTGTACAGAGATGA	AGACACTGACGCCCATGAAT
OsCSLC1 qPCR	GGGGGTTACAATTCATCGGAGA	AAACAACCCATTCTAACCACTGAG
OsCSLC7 qPCR	AAGAGTGATGCAAATGTTCGATG	ATCTATCTACATCTCCACAGTTCAGT
OsCSLC9 qPCR	ACAGTGACAATGGAGGGTGCT	GGGCGTGTACATTGTGGATCAT
OsCSLD2 qPCR	CAAGGGGCTAATGGGAAGGAG	GGCAACCCACAGCAATGAGA
OsCSLE1 qPCR	GTGTTTTTACCCCAGGCCATC	TGACTGCTGTTGGTATTCTCCC
OsCSLF6 qPCR	CCGGAGACGAAGAAGAAAACACA	GTTGCAGCAGCGTGTAGTAGAA
OsCSLH1 qPCR	AGAATCTACTCGTCCATGGCAAG	ACCGCTCCAATGCTTCTACTTT
OsBRD2 qPCR	GAGGCGTAATTTCGTTGAGACC	CGATGACAGGATTACAAAGTGCTAC
OsD2 qPCR	GCCACCACTACTACTATACCGATC	TCGTGTGGGCTACTCGTACT
OsD11 qPCR	GGTGTAGATATATTTTGTCCATGCCG	AGCAGATGAAAGTTGAAACAGTGG
OsDWARF4 qPCR	GAGCCTTTTGACCTAATTGTTGGA	CGAAAACGTGTACATGCATCCT
OsBRD1 qPCR	GATGACAGGATTAGAACAGCCG	GATGGACCAAAAGAATACAGGAGC
OsBRI1 qPCR	CTCCTCATCACTTCCCACTCTCC	AGCTCACTGCCTCACGACC
OsBAK1 qPCR	CTCAACTCAACCCCCCCCAA	GATCCCCTTCTCTCGCTTTTCG
OsBSK3 qPCR	CATGCCCTTGACCTGATTCGAG	TCGCACTAGTTCTGTCCCTTCC
OsGSK1 qPCR	CTAAGTGCTTGGAGACGGGG	AGGCAGATGACATTGGGGTG

OsGSK2 qPCR	AGACCTTTTGTTGGATCGTTTTCG	TTCTTCTTGTTTTGCGGGGATTC
OsBZR1 qPCR	GCCGAGCAAAAAAGATGGTTCC	GAATGAAATCGCCCAAATCGCA
OsLIC qPCR	GTTGCCACCATATGTCGTACTTT	CACTGTTCACTCTTGCAAATTCTCT
OsDLT qPCR	GGCTGTTGAGAGAGAGTCCC	ATAGTGACTGTGAGAGATGCTGC
OsRLA1/SMOS1 qPCR	ATCCTGCACGTCTATGGGTTTC	ACTTCTCTTACCTTCTATTCATGGCT
OsD1/RGA1 qPCR	AAGTCACACAGGGAAGGTAATTAGG	CAAAAGATCAATCAATGGTCCACGT
OsTUD1 qPCR	GAGTGGAGTTTGGAATTGTGCTG	GCTGTCGACTGCCAATTGCTA
OsBU1 qPCR	GGATGATATGAATGCAGCTCGT	ATCATCATCATCAGTAGTACACCG
OsTAA1 qPCR	TTGTAAGTTGAAGTCCTGCCA	AAAACACAACATCGAGAAACAA
OsYUCCA3 qPCR	AGCTAGTAGGTTTGGTGGTGATA	CGCACGCAATTACACCCTTT
OsYUCCA4 qPCR	GCTGGTCTGGTTTACATTTCA	GGGGAAAAAAATGAGATGCAC
OsYUCCA5 qPCR	GTTCTGTCGTCGCTGTGTT	GATTGATCTCATTCTCGACCAGC
OsYUCCA6 qPCR	ACATTGATGAGGAGGCCAGA	CATTCTGTACATAAAGGCCCAT
OsYUCCA7 qPCR	ACCGTTGCTACTGCTACTC	GTATGATCACACTCTCCTAGCTT
OsGH3-1 qPCR	GCAATGGAACAAAAGCAAGGA	CAGATCATCACCCTCTAGCTTCAA
OsGH3-3 qPCR	CCTCCTAATGACGATCTGTCCTATG	GGATTCCGAGCTGCTGATAAGA
OsGH3-4 qPCR	AGAGAGAATTTGCTAGCTATGGTGATT	CTACCCTGAACTAACACTCGTTGATTA
OsGH3-6 qPCR	CACTAGCATCTGTCTCATTGTGTCA	ACCTTGTCAGTGCCGGAATT
OsGH3-7 qPCR	ATTGGAGGAAAGGGTTGTAGGAA	CTCCTCTCAAATTCGGCTGAA
OsDAO qPCR	ATGCTGAGGGGATGGGAATT	CTCCACCTTCCAGTTATTCCTAC
OsCycD4;2 qPCR	GGGGCCAAAAGGGAGGGAAT	CTTTGGGGGCACCTCTCATCA
OsCycD6;1 qPCR	AGGCAACGTGAGCGAGAGATAG	AGAAAGAGCAGGGCAAGAGCAA
OsCycH1;1 qPCR	AGGTGAGGTTCCGTTTTAGCC	CACAAGATTACAAGAAGGCGAGG
OsCycU1;1 qPCR	GAGCGCGTTCTGTGTGTATATATC	CACTTTGGCAGGTAATTAATCCGTC
OsCycU3;1 qPCR	CAAGTTCACGGCGTCAATAGCA	TACCGCATCAGCTCGCTCTT
OsCycU4;1 qPCR	AGCTCTTCTTTTTTGTCACTGGA	AGCTCAATTCTTTCACTAACCACA
OsCDKA;2 qPCR	TAAGTTGGTGTGCTCCTCCC	CAGAGGGATAGCCAGGAGGTA
OsCDKB;1 qPCR	GTTAGCAGCAAGGAAATTCGTT	CCATACAGCATAGAAACAAACCC
OsCDKC;2 qPCR	GCGGTATCATGAGCAGCAAAT	ATACTTCCGAGAGATGCCTGG
OsCDKD;1 qPCR	CATAGATGAGTTTGCTTGTGTGAG	CACCAGAAAGAACTTGAGACATAAAC
OsCDKE;1 qPCR	ATTTGGTGTGTTACTTGTGAGC	CAATTCTCACTGTACGACTAGGA
OsCDKF;1 qPCR	ATGGGCTAATTTCAGGGGTGG	AAATTCCTCTGCCAGCATGTTC
OsEXPA1 qPCR	AAGTTTGGAGCATGCGCGC	CAAGCACCTCGCAAACTGTACA
OsEXPA4 qPCR	TCGTCGTCGTCTTTCTTCCTT	TCAGCATAGCCCAAAACCTCT
OsEXPA6 qPCR	AGTAGATGCTGAGGTTGCTGTG	AACTAAGAGCAGAGCAGCAAACT
OsEXPA10 qPCR	AGAAATGTTTAGTGCAGAGC	GTAGTGCAATTATCAGAGTTCC
OsEXPB3 qPCR	ATTTGAGATCGATCGTTTGGC	CAAAACGAACTCCTGATGACA
OsEXPB4 qPCR	GGGTTCTTTGAGTTTGTTGGGG	CCTCCTCCATTTCCCACACAG
OsEXPB6 qPCR	CTGGTCTAGTGTGTGAAGT	CTGTAGCCTTAAGATTTGGTT
OsEXLA2 qPCR	CATGTACAGTTTTTTTGCTTTCCTTT	CTGTGGCTCTTAATTCGATGGAT
OsEXLA3 qPCR	GACGTATCTGGCACTGCAAAG	GTTTTGACAGGGAAGGGGAGC
OsXTH9 qPCR	CCCGAGTGCTCCATGCCGTA	TGAGTTCGATCGAGTCCGCGTTC
OsXTH10 qPCR	CCCCTGAATCTCCCACACAC	GATCAATGGGGGAGCTCGAA
OsXTH11 qPCR	ACACGAGAAGATGATCATAACG	ATTATTCGCCATCTGACGTG
OsXTH12 qPCR	TCTGTATCTATTGCTGTATGTG	CTTATTATACATCCATCGTCGT
OsXTH15 qPCR	CTCTGGTTGTATGCTCCGGAC	CCGATGGACGATGTGTAGTAGG
OsXTH17 qPCR	GATGAGTTCTTGGCAATGATT	AACAAATGGAGAACGAATGGA
OsXTH21 qPCR	CCCGAATGCTGAGCTACTTTT	ACACAACACACACGAATCAGC
OsXTH23 qPCR	TTGGGAAGCTAATGGCATATG	GCACCTACTACCTTTACAAAC
OsXTH28 qPCR	ATTCCGCGCTCTGACCGAT	CCAGGAAACTATAATCACGTACGAC
